# Root Niches of Blueberry Imprint Increasing Bacterial-Fungal Interkingdom Interactions along the Soil-Rhizosphere-Root Continuum

**DOI:** 10.1128/spectrum.05333-22

**Published:** 2023-05-24

**Authors:** Jilu Che, Yaqiong Wu, Hao Yang, Shaoyi Wang, Wenlong Wu, Lianfei Lyu, Xiaomin Wang, Weilin Li

**Affiliations:** a Co-Innovation Center for Sustainable Forestry in Southern China, Nanjing Forestry University, Nanjing, China; b Institute of Botany, Jiangsu Province and Chinese Academy of Sciences (Nanjing Botanical Garden Mem. Sun Yat-Sen), Jiangsu Key Laboratory for the Research and Utilization of Plant Resources, Nanjing, China; Nanjing Institute of Geography and Limnology, Chinese Academy of Sciences

**Keywords:** compartment niches, root-associated microbiome, rhizosphere, bacterial and fungal communities, co-occurrence networks

## Abstract

Plant root-associated microbiomes play critical roles in promoting plant health, productivity, and tolerance to biotic/abiotic stresses. Blueberry (Vaccinium spp.) is adapted to acidic soils, while the interactions of the root-associated microbiomes in this specific habitat under various root microenvironments remain elusive. Here, we investigated the diversity and community composition of bacterial and fungal communities in various blueberry root niches (bulk soil, rhizosphere soil, and root endosphere). The results showed that blueberry root niches significantly affected root-associated microbiome diversity and community composition compared to those of the three host cultivars. Deterministic processes gradually increased along the soil-rhizosphere-root continuum in both bacterial and fungal communities. The co-occurrence network topological features showed that both bacterial and fungal community complexity and intensive interactions decreased along the soil-rhizosphere-root continuum. Different compartment niches clearly influenced bacterial-fungal interkingdom interactions, which were significantly higher in the rhizosphere, and positive interactions gradually dominated the co-occurrence networks from the bulk soil to the endosphere. The functional predictions showed that rhizosphere bacterial and fungal communities may have higher cellulolysis and saprotrophy capacities, respectively. Collectively, the root niches not only affected microbial diversity and community composition but also enhanced the positive interkingdom interactions between bacterial and fungal communities along the soil-rhizosphere-root continuum. This provides an essential basis for manipulating synthetic microbial communities for sustainable agriculture.

**IMPORTANCE** The blueberry root-associated microbiome plays an essential role in its adaptation to acidic soils and in limiting the uptake of soil nutrients by its poor root system. Studies on the interactions of the root-associated microbiome in the various root niches may deepen our understanding of the beneficial effects in this particular habitat. Our study extended the research on the diversity and composition of microbial communities in different blueberry root compartment niches. Root niches dominated the root-associated microbiome compared to that of the host cultivar, and deterministic processes increased from the bulk soil to the endosphere. In addition, bacterial-fungal interkingdom interactions were significantly higher in the rhizosphere, and those positive interactions progressively dominated the co-occurrence network along the soil-rhizosphere-root continuum. Collectively, root niches dominantly affected the root-associated microbiome and the positive interkingdom interactions increased, potentially providing benefits for the blueberry.

## INTRODUCTION

Plant root-associated microbiomes are essential to the health and productivity of host plants, providing multiple benefits to host plants, including nutrient uptake, growth promotion, resistance to pathogens, and stress tolerance (1, 2). Furthermore, microbiomes can contribute to soil health and environmental sustainability by promoting soil nutrient turnover and maintaining soil fertility, such as by facilitating carbon and nitrogen cycling in ecosystems by decomposing soil organic matter and fixing atmospheric nitrogen (3–5). Thus, rational utilization of root-associated microbiomes is increasingly considered for sustainable agricultural production (6–8). Moreover, the antagonistic, competitive, or mutualistic interactions between the microbes activate the energy flow and nutrient cycles in underground microecosystems, playing a basic role in shaping and structuring complex microbial networks (8). By providing novel metabolic capabilities to their microbial associates, host plants contribute to niche-specialized inhabitant adaptation, which can influence soil function and plant productivity (9). Therefore, understanding the taxonomic and functional components and the interactions among microbial communities along the soil-rhizosphere-root continuum is critical for the precise manipulation of beneficial microbiota to achieve sustainable ecosystem functions (10, 11).

Blueberry (Vaccinium spp.) is an agricultural crop cultivated worldwide for its high economic value. Its fruits contain high amounts of phenols and anthocyanins, which have beneficial effects in maintaining blood sugar levels, reducing oxidative stress, and preventing cardiovascular diseases, as well as anti-inflammatory, antimicrobial, and antitumor activities (12, 13). Although the shallow root systems and sparse root hairs limit the efficient uptake of water and nutrients, blueberry plants are well adapted to acidic soil conditions (14, 15). This adaptation could be associated with the establishment of beneficial interactions with soil microorganisms (16). Thus, revealing the contribution of microorganisms in the adaptation of blueberry to acidic soils could provide a basis for harnessing microorganisms to promote the adaptation of blueberry to various soil conditions to improve yield and fruit quality and reduce chemical inputs. A recent study showed that blueberry rhizosphere-isolated strains with auxin production, phosphorus solubilization, and nitrogen fixation capacities can increase the germination rate of blueberry seeds, which is considered helpful in promoting the growth of blueberry (17). In addition, ericoid mycorrhizal (ERM) fungi can form symbiotic relationships with blueberry roots that can play a critical role in the survival of plants growing in habitats with low soil pH and slow organic matter turnover by secreting a broad range of enzymes, including cellulases, proteases, polyphenol oxidases, and phosphatases, which decompose complex organic compounds and enhance the fitness and nutrient acquisition of the host plant (18–20). Several studies have focused on the impact of host cultivars (21–23), differential habitats (16, 24, 25), and agricultural practices (26), but further exploration of the interactions of blueberry root-associated microorganisms along the soil-rhizosphere-root continuum is needed.

Plant health and productivity are intimately associated with the microbial communities that inhabit the soil-rhizosphere-root continuum (27). As influenced by plant root activity, the diversity and composition of the root-associated microbiome transition from the external to the internal part of the plant root system, shaping specific plant root compartment niches in terms of the bulk soil, the rhizosphere, the rhizoplane, and the endosphere (28, 29). The bulk soil, which has high microbial diversity, serves as a reservoir, providing specific microorganisms for each compartment niche. The rhizosphere and rhizoplane microbial communities are shaped by the metabolic activities of plant roots, and a subset of rhizosphere microbes penetrate the plant roots and colonize the endosphere, which is subject to the plant immune system. Ultimately, each compartment niche develops a microbial community with distinctive structural characteristics (29–31). The diverse microbial communities in each ecological niche of the root system are influenced by complex factors associated with the assembly process. Thus, the assembly process provides important insights for discovering the differences in community composition, and quantifying these processes is vital to elucidate the mechanisms governing microbial community structure (32). Microbes can be inherited by vertical transmission from seeds and can also colonize various plant compartment niches via air, soil, and nearby plants, forming a dynamic community in response to the combined effects of the host plant and environmental factors (33–35). Plant microbiome assembly develops with plant growth and is influenced by deterministic and stochastic processes, such as biotic- and abiotic-factor-mediated selection or random drift and dispersal events, respectively (9, 36). The assembly process of plant root-associated microbes is recruited through a two-step selection mode and is driven by many factors, such as edaphic factors and plant root metabolites and exudates (37, 38). However, we still lack a comprehensive understanding of the assembly processes of the blueberry root-associated microbiome, which is intimately related to the network of plant-microbe interactions.

Microbial co-occurrence network analysis allows the characterization of potential microbial interactions in various habitats (39, 40), specifically to visualize the response patterns of taxonomic groups to various plant compartment niches and to identify keystone microbes that significantly shape community composition (6, 41). For instance, the average degree and modularity of topological characteristics present the connectivity and complexity of members in co-occurrence networks (42). The interactions between bacteria and fungi, with dynamic changes, are intimately related to plant growth and development. A recent study revealed that interactions between oomycetes, fungi, and bacteria can contribute greatly to plant survival and that bacterial communities play an essential role in maintaining a balanced interkingdom network by protecting plants against pathogens (43). Furthermore, the interkingdom interactions between bacteria and fungi clearly shift during different stages of plant development, and microbial network hubs play a crucial role in maintaining plant fitness and nutrition during plant growth (39). Therefore, specific microbes that inhabit different compartment niches and may have important ecological functions in the mutually beneficial symbiotic relationship between plants and microbes can be tapped via a co-occurrence network.

In this regard, we aimed to explore the variable characteristics of the blueberry root-associated microbiome along the soil-rhizosphere-root continuum, as well as microbial assembly processes and co-occurrence networks. The diversity and community composition of the bacterial and fungal communities in the bulk soil, rhizosphere soil, and root endosphere were explored in these three root compartment niches of three host cultivars, rabbiteye blueberry, northern highbush blueberry, and southern highbush blueberry. We hypothesized that the interkingdom interactions of bacterial and fungal communities would increase along the soil-rhizosphere-root continuum. Hence, our objectives were to (i) determine how blueberry root-associated microbial community composition differs across root compartment niches, (ii) elucidate the assembly processes in each compartment niche, and (iii) explore the interkingdom network patterns along the soil-rhizosphere-root continuum.

## RESULTS

### Diversity and abundance of root-associated microbial communities.

**FIG 1:**
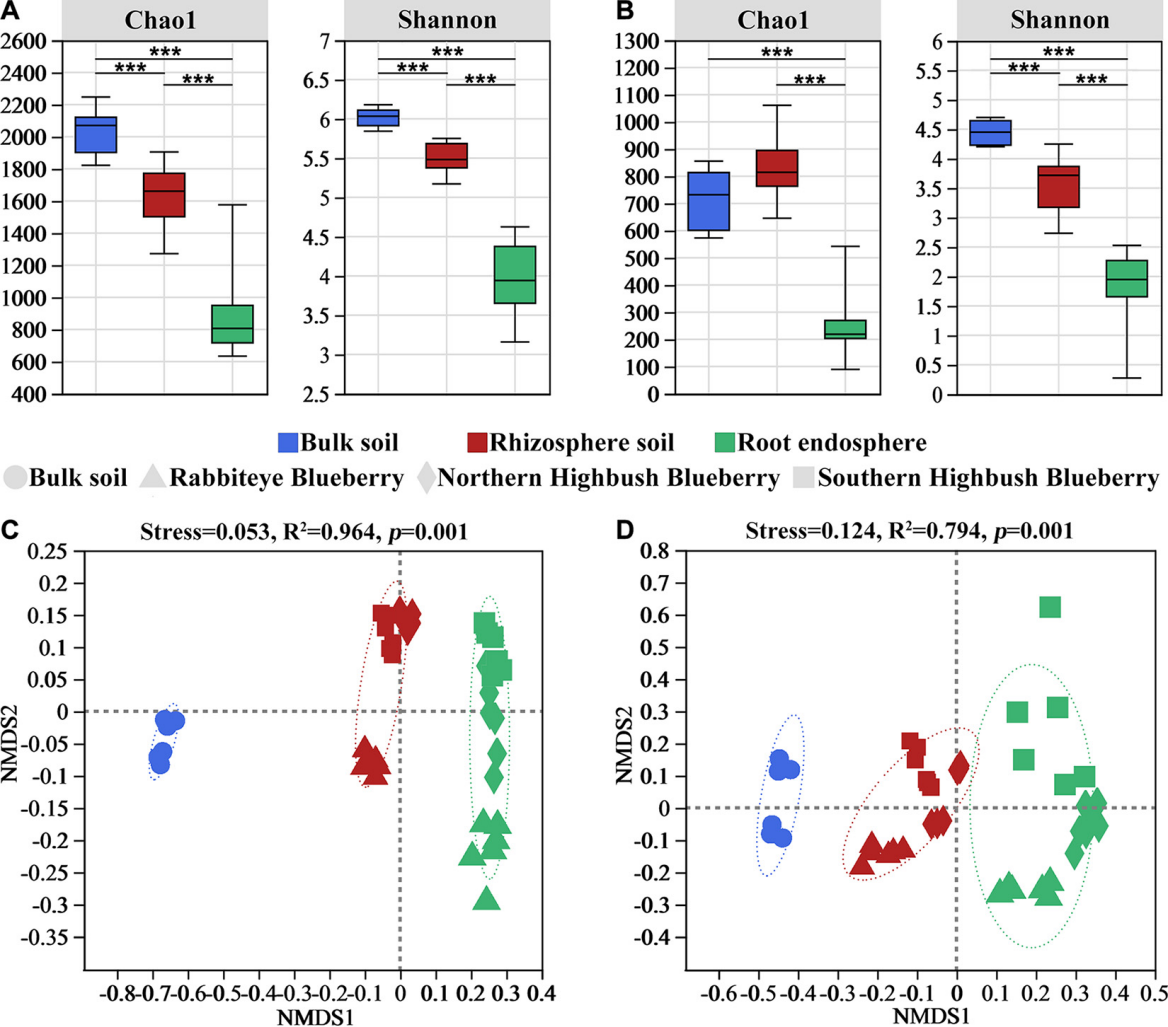
(A and B) Alpha diversity (Shannon and Chao1 indices) of bacteria (A) and fungi (B) in different compartment niches of blueberry. Significant differences between the bulk soil, rhizosphere soil, and root endosphere are indicated in each figure panel: *, *P < *0.05; **, *P < *0.01; ***, *P < *0.001. (C and D) NMDS analysis of bacterial (C) and fungal (D) communities along the soil-rhizosphere-root continuum of blueberry based on Bray-Curtis distance metrics.

The Chao1 and Shannon indices of the bacterial and fungal communities were significantly higher in the bulk soil than in the rhizosphere soil and root endosphere, except for the Chao1 index of the fungal community in the rhizosphere soil (Fig. 1A and B). The root compartment niches clearly had significant effects on the alpha diversity of the bacterial and fungal communities and showed a decreasing trend along the soil-rhizosphere-root continuum of blueberry. Nonmetric multidimensional scaling (NMDS) ordinations and two-way permutational multivariate analysis of variance (PERMANOVA) indicated that the variations in both bacterial and fungal communities were mainly explained by the root compartment niches (*R*^2^ = 0.564, *P < *0.001; *R*^2^ = 0.339, *P < *0.001) and then by the host cultivars (*R*^2^ = 0.161, *P < *0.001; *R*^2^ = 0.182, *P < *0.001) (Fig. 1C and D; Table S1 in the supplemental material). In addition, both bacterial and fungal communities were clustered into three groups, and analysis of similarity (ANOSIM) tests (Fig. 1C and D) showed significant differences in the taxonomic compositions of these three compartment niches. These results indicated that the compartment niches had strong effects on the diversity and abundance of bacterial and fungal communities compared to those of the host cultivars of the blueberry plants.

### Compositions of the root-associated microbial communities.

**FIG 2:**
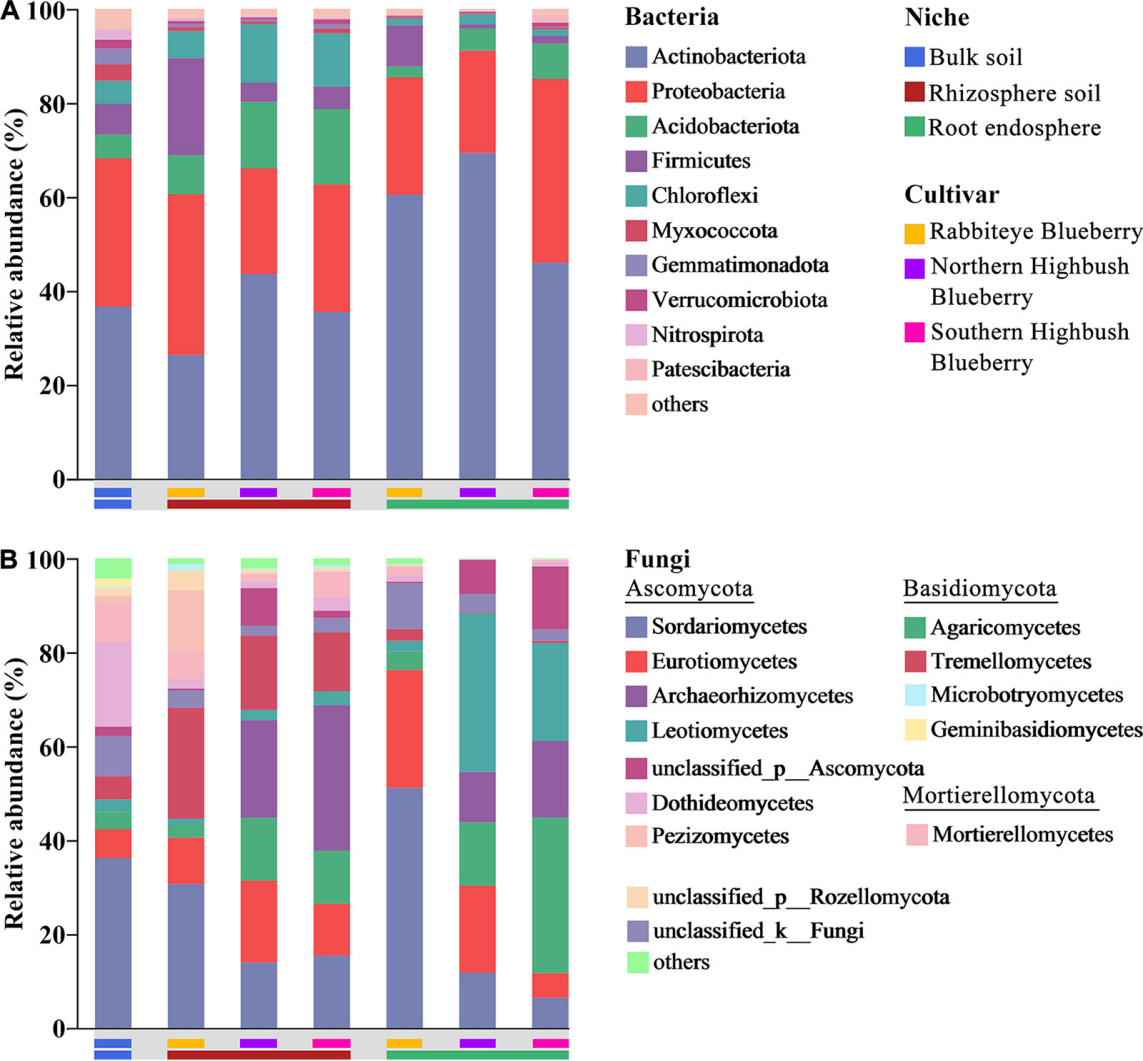
Taxonomic compositions of bacterial (phylum level) (A) and fungal (class level) (B) communities of blueberry in different compartment niches.

The compositions of the bacterial and fungal communities varied among the three root compartment niches. *Actinobacteria* (36.7%, 35.1%, 58.6%) and *Proteobacteria* (31.5%, 28.0%, 28.6%) were the predominant phyla in the bacterial community composition of the three compartment niches, with *Actinobacteria* being more abundant in the root endosphere and *Proteobacteria* being more abundant in the bulk soil (Fig. 2A). The root compartment niches also altered the low-abundance phyla, with *Myxococcota*, *Gemmatimonadota*, *Verrucomicrobiota*, and *Nitrospirota* being the most abundant in the bulk soil and decreasing along the soil-rhizosphere-root continuum of blueberry roots. Interestingly, the low-abundance phyla *Acidobacteria*, *Firmicutes*, and *Chloroflexi* were more abundant in the rhizosphere. For fungal communities, *Sordariomycetes*, *Dothideomycetes*, and *Mortierellomycetes* were significantly enriched in the bulk soil, with *Sordariomycetes* being the most abundant class, and significantly reduced in both the rhizosphere and endosphere. *Archaeorhizomycetes*, *Tremellomycetes*, and *Pezizomycetes* were significantly enriched in the rhizosphere, while *Eurotiomycetes*, *Agaricomycetes*, and *Leotiomycetes* were more abundant in the root endosphere (Fig. 2B). Furthermore, the ERM fungi *Helotiales* (2.3%, 1.8%, and 19.0%), *Chaetothyriales* (1.4%, 7.2%, and 15.0%), and *Sebacinales* (0%, 7.1%, and 5.4%) were observed in the three compartment niches and were significantly enriched in the root endosphere (Fig. S1).

### Biomarkers of bacteria and fungi in the three compartment niches.

**FIG 3:**
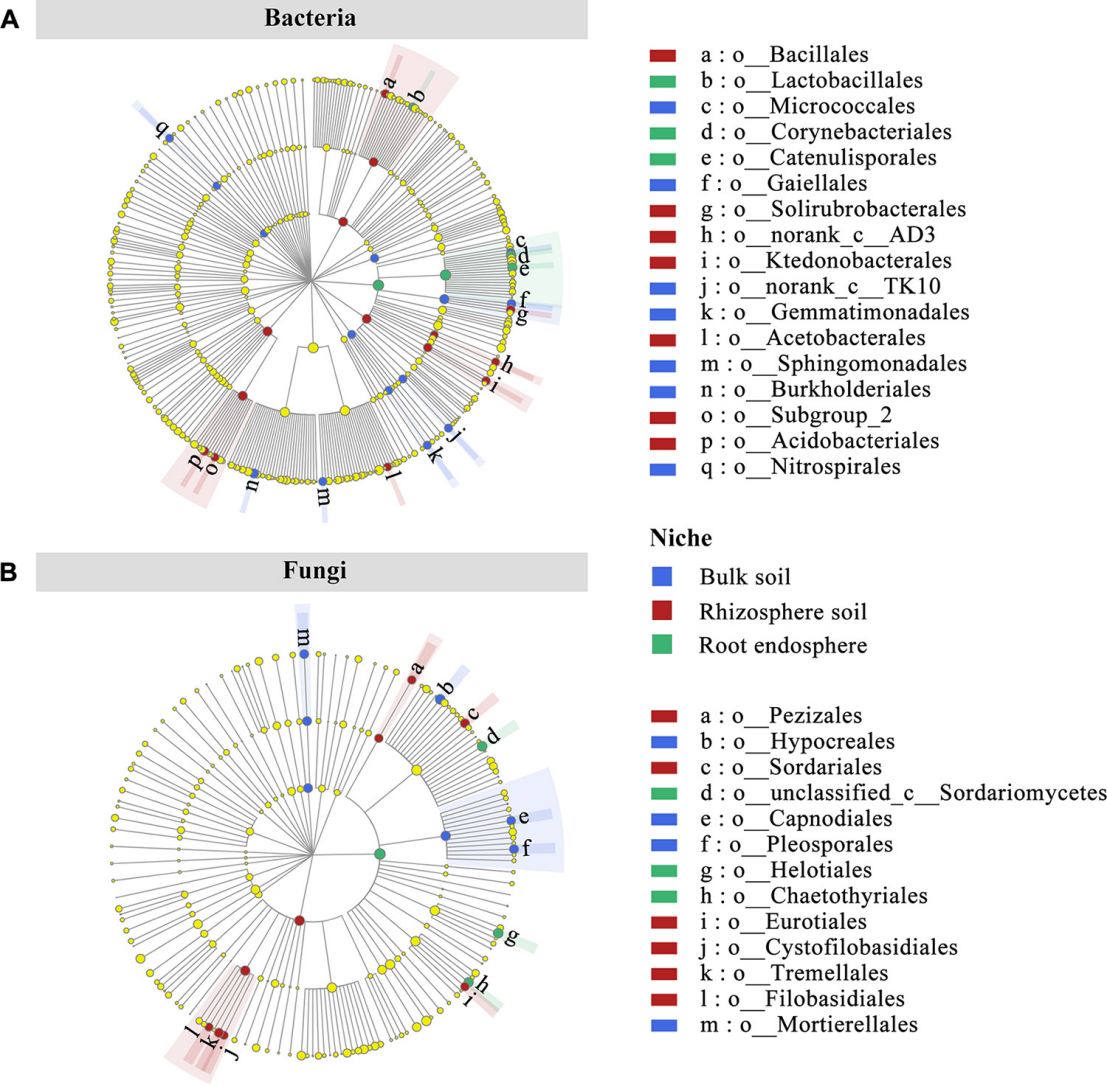
LEfSe analysis of differentially abundant (LDA threshold score of ≥4.0) taxa from phylum to order of bacterial (A) and fungal (B) communities of blueberry among different compartment niches.

The distinct taxa of the three compartment niches were evaluated by linear discriminant analysis (LDA) effect size (LEfSe) scores at the phylum, class, and order levels. A total of 33 distinct bacterial biomarkers were identified (LDA ≥ 4.0, *P < *0.05), including 7, 9, and 17 biomarkers of phylum, class, and order, respectively, mainly associated with the phyla *Firmicutes*, *Myxococcota*, *Actinobacteria*, *Chloroflexi*, *Gemmatimonadota*, *Acidobacteria*, and *Nitrospirota* (Fig. 3A; Fig. S2). A total of 20 fungal biomarkers, including 3, 4, and 13 biomarkers of phylum, class, and order, respectively, were distributed among different niches. These biomarkers were associated with the classes *Mortierellomycetes*, *Pezizomycetes*, *Dothideomycetes*, and *Tremellomycetes* (Fig. 3B; Fig. S2).

### Assembly processes of bacterial and fungal communities.

**FIG 4:**
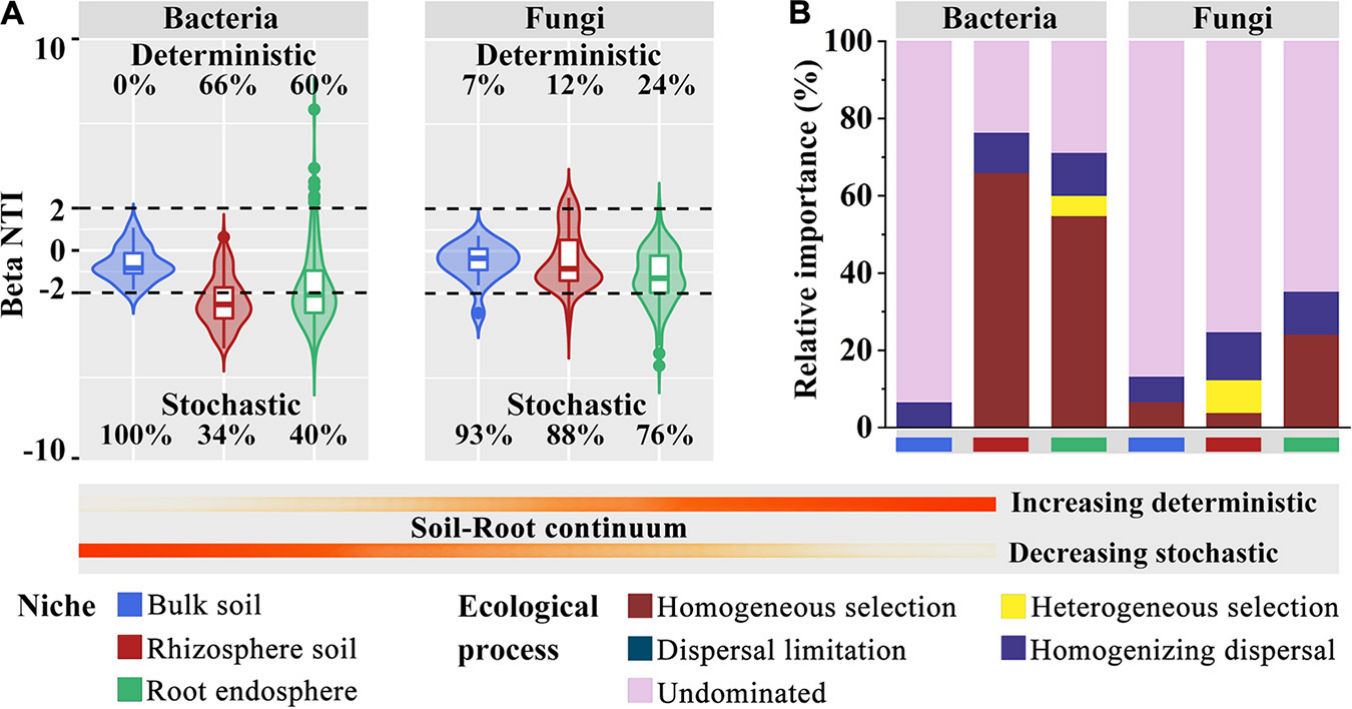
Assembly processes of bacterial and fungal communities. (A) Relative contributions of deterministic and stochastic processes in microbiome assembly based on the βNTI values. The percentages above and below the violin plots represent the proportions of the deterministic processes and stochastic processes in the microbiome assembly, respectively. (B) Relative importance of five ecological processes based on both βNTI and Bray-Curtis-based Raup-Crick index (RC_Bray_) values.

To explore the process of microbiome assembly among the various compartment niches of blueberry roots, we performed a null model analysis. Stochastic processes were the predominant drivers of both bacterial and fungal communities in the bulk soils (Fig. 4A). The relative contributions of deterministic processes belonging to homogeneous selection were higher in the rhizosphere (66%) and endosphere (55%) bacterial communities, whereas higher relative contributions of stochastic processes belonging to undominated processes were observed in the rhizosphere (75%) and endosphere (65%) fungal communities (Fig. 4A and B; Table S2). It was clear that deterministic processes increased and stochastic processes decreased along the soil-rhizosphere-root continuum for both bacterial and fungal communities in the blueberry roots. Collectively, deterministic processes had a greater influence on the assembly processes of bacterial communities in the rhizosphere and endosphere, while stochastic processes were the dominant assembly processes of fungal communities. In particular, the assembly of bacterial communities in the rhizosphere and endosphere was more strongly dominated by deterministic processes than that of fungal communities.

### Bacterial-fungal community interkingdom co-occurrence networks.

**FIG 5:**
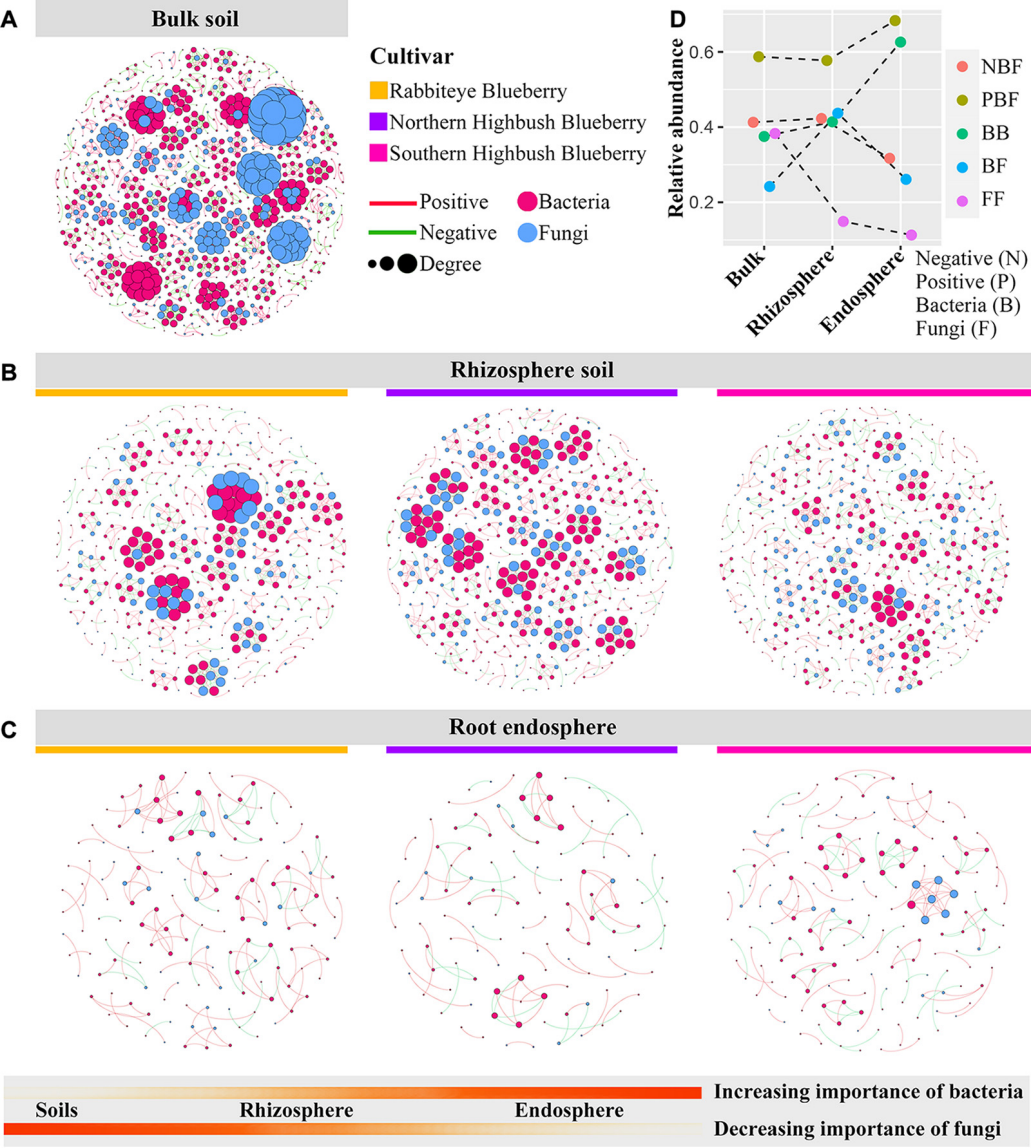
(A to C) Co-occurrence network analysis showing the different bacterial-fungal interkingdom network patterns in the bulk soil (A), rhizosphere soil (B), and root endosphere (C) of blueberry. (D) The relative abundances of multiple correlations between bacterial and fungal taxa in interkingdom networks in different compartment niches. Connections indicate significant (*P < *0.01) correlations, which were divided into positive (Spearman’s *P > *0.7; red) or negative (Spearman’s *P* < −0.7; green) correlations.

We further performed a co-occurrence network analysis to assess the bacterial-fungal interkingdom interactions among root compartment niches. The relative proportions of bacterial nodes and correlations increased consistently from the soils to the endosphere, while fungi showed the opposite trend. The average degree in the bacterial-fungal interkingdom co-occurrence network decreased from the bulk soil to the endosphere (Fig. 5; Table S3). In the bulk soil, the average degrees were much higher in fungal communities than in bacterial communities, with average degrees of 7.961 and 4.169, respectively (Fig. S3 and Tables S3 and S4). Bacterial-fungal interkingdom interactions were clearly high in the rhizosphere, while their proportions of positive interactions increased from the soil to the endosphere (Fig. 5D; Table S6). Hence, the different compartment niches of blueberry roots markedly influenced the bacterial-fungal interkingdom network interaction patterns, and positive bacterial-fungal interkingdom interactions gradually dominated in the co-occurrence networks along the soil-rhizosphere-root continuum. Several keystone taxa with the highest node degrees were identified in the bulk soil, rhizosphere, and endosphere, which mainly belonged to the phyla *Proteobacteria*, *Actinobacteria*, *Acidobacteria*, and *Chloroflexi* in the bacterial communities (Table S7) and the classes *Eurotiomycetes*, *Leotiomycetes*, *Sordariomycetes*, and *Agaricomycetes* in the fungal communities (Table S8).

### Functional profiles of microbial communities in different compartment niches.

**FIG 6:**
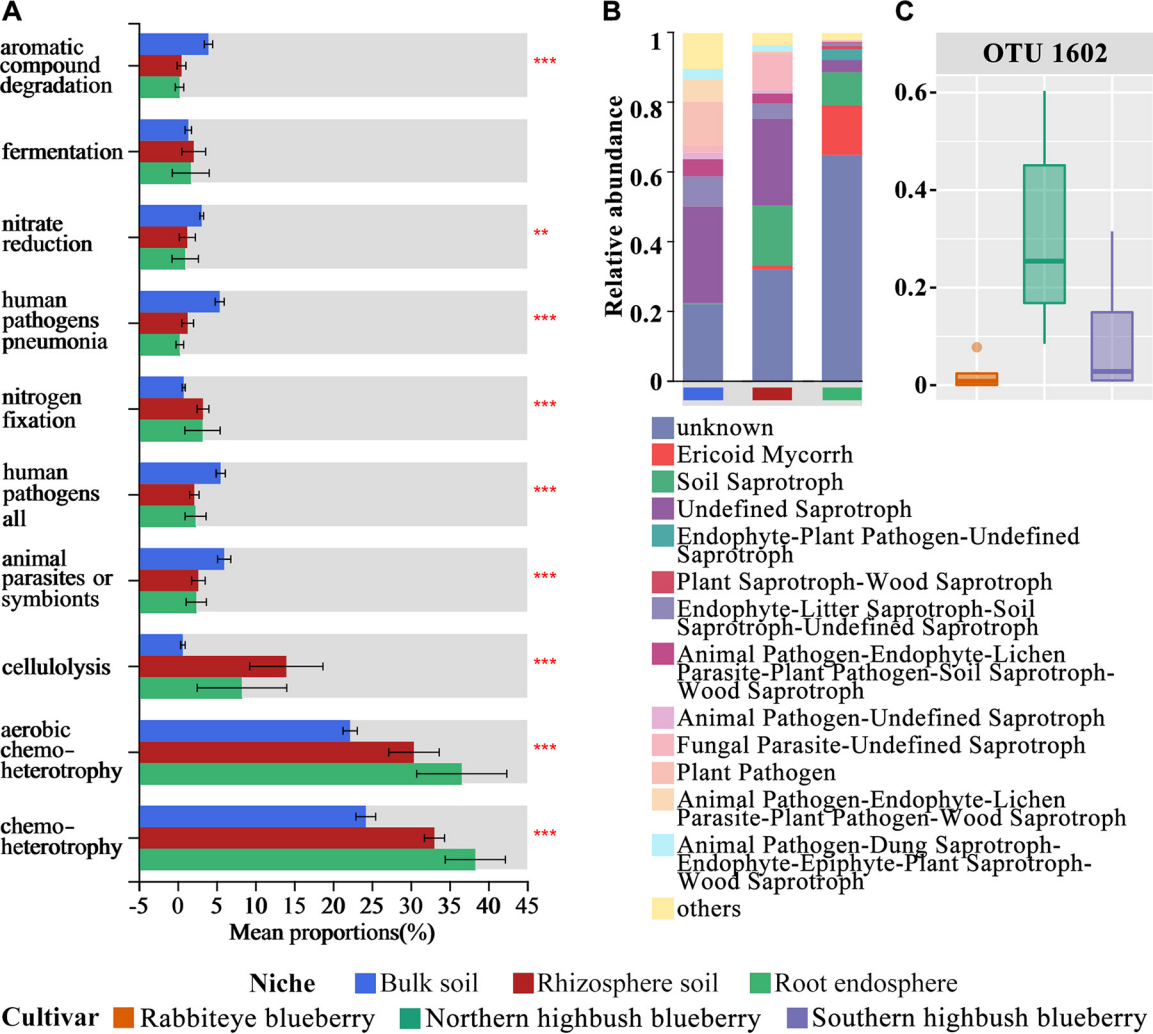
(A and B) Functional predictions of bacteria (A) and fungi (B) in the root compartment niches of blueberry. The top 10 functional abundances of the bacterial community are shown, with the vertical axis indicating the functional names and the horizontal axis indicating the percentage values of the different functional abundances. Error bars show standard deviations. *, 0.01 < *P < *0.05; **, 0.001 < *P < *0.01; ***, *P < *0.001. The horizontal axis in the fungal function prediction results indicates the different root compartment niches, and the vertical axis indicates the relative abundances of different functions in each niche. (C) Relative abundances of one taxon in root microbiomes of three cultivars.

The proportions of the top 10 functions in the bulk soil, rhizosphere, and endosphere were 73.62%, 91.00%, and 94.59% of the overall functionality, respectively (Fig. 6; Fig. S4 and Table S9). The bacterial community mainly performs the functions of chemoheterotrophy and aerobic chemoheterotrophy in each compartment niche, specifically in the rhizosphere and endosphere. The bacterial communities in the rhizosphere and endosphere showed stronger capacities for cellulolysis and nitrogen fixation than for aromatic compound degradation. Notably, the relative abundance of cellulolysis-associated microbes was significantly higher in the rhizosphere than in the bulk soil and endosphere (Fig. 6A). The main ecological guild varied significantly among the different compartment niches; the highest relative abundance of ericoid mycorrhizae was in the root endosphere (14.1%), that of soil saprotrophs was in the rhizosphere (17.3%), and a high relative abundance of plant pathogens was in the bulk soil (12.4%) (Fig. 6B). In particular, operational taxonomic unit 1602 (OTU1602) from the ecological guild of ericoid mycorrhizae was identified, and its relative abundance varied among the three cultivars (Fig. 6C). The relative abundance of plant pathogens decreased along the soil-rhizosphere-root continuum from the bulk soil to the rhizosphere to the endosphere by 12.4%, 0.8%, and 0.1%, respectively.

## DISCUSSION

### Root compartment niches shape diverse microbial communities.

Different plant compartment niches harbor specific microbiomes (31, 42). Our results indicated that root compartment niches dominantly shaped the root-associated microbial communities, with decreasing alpha diversity along the soil-rhizosphere-root continuum. The gradual shifts in microbial communities include enrichment and depletion processes from the surrounding soil microbiota (30). Previous studies have shown that plants can alter microbial communities by secreting bioactive molecules, including primary metabolites and secondary metabolites, into the rhizosphere (44). The interaction between microbial substrate uptake and plant exudative traits forms a molecular mechanism to regulate microbial communities, such as the recruitment of the colonization of bacterial communities that prefer aromatic organic acids by regulating the content of aromatic organic acids (45). In addition to providing typical carbon and nitrogen substrates, roots actively regulate the rhizosphere microbial community by secreting specific chemicals (46). Since microbial communities with the ability to promote growth and activate induced systemic resistance are highly tolerant of coumarins, plants can participate in shaping the rhizosphere microbiome by regulating coumarin-biosynthetic pathways (47). The rhizosphere microbiome acts as an important inoculum reservoir and, thus, contributes to the construction of the endosphere microbial community (48). The host immune system has a strong influence on the enrichment and depletion processes of the microbial community from the rhizosphere to the endosphere, and only a fraction of the microbiota in the rhizosphere with traits that subvert the host immune processes have the potential to colonize the endosphere (30). Consequently, this leads to differentiation of microbial communities in various compartment niches.

Furthermore, host selection effects are enhanced along the soil-rhizosphere-root continuum with the recruitment, filtration, and enrichment of microbial taxa with specific functions in different compartment niches. In contrast to environmental factors, such as fertilization practice or site, host selection dominates in shaping the assembly of microbial communities, primarily through compartment niches and then host species (2). This was also evident in our study, where variations in the taxonomic compositions of blueberry root microbiomes were dominated by the root compartment niches rather than by the host cultivars (Fig. 1B; Table S1). Studies on Zea mays, Triticum aestivum, and Hordeum vulgare show that different species have an effect on the composition of the microbiomes (2), while different genotypes of Arabidopsis thaliana show that different host genotypes have a limited effect on the root microbiomes (49, 50). Although different host plant subspecies, cultivars, and genotypes can harbor distinct microbial communities, within individual species, this effect typically appears rather weak (31, 51, 52). Therefore, the root compartment niche is the primary driver shaping the composition of root-associated microbial communities, rather than the blueberry cultivar. The varied compartment niches affected the proportions of the root-associated microbial community composition of blueberry. Consistent with previous studies, *Actinobacteria* and *Proteobacteria* were the most abundant phyla in the bacterial community (22), and our results showed that *Actinobacteria* was the dominant phylum in the endosphere. Members of *Actinobacteria* possess a potential suppressive effect on plant pathogens (53), and the increased proportion in the endosphere may have been related to the selection of functions that correspond to plant health requirements. Moreover, the increased proportions of *Firmicutes* and *Chloroflexi* in the rhizosphere indicated a potential dominant function in nutrient cycling, as these microorganisms are related to carbon and nitrogen cycling (54, 55). Thus, the different community compositions of each compartment niche were likely related to their functional requirements for biotic stress reduction or nutrient cycling. Similarly, the fungal communities showed compartment-specific enrichment patterns, with high proportions of *Archaeorhizomycetes* and *Tremellomycetes* in the rhizosphere and high proportions of *Eurotiomycetes*, *Agaricomycetes*, and *Leotiomycetes* in the endosphere (Fig. 2B). Members of *Archaeorhizomycetes* are considered to have saprotrophic potential and are dependent on root-derived carbon compounds (18), which may be attributed to the active root metabolism that enhances their assembly in the rhizosphere. Due to the poor root system of blueberry plants, they can be symbiotic with ERM fungi to cope with their limitations in nutrient uptake and adaptation to low soil pH (56). Therefore, the high relative abundances of ERM fungi belonging to *Eurotiomycetes*, *Agaricomycetes*, and *Leotiomycetes* in the endosphere were likely to be potentially beneficial in fulfilling root nutrient requirements.

### Dynamic ecological roles of bacterial and fungal communities along the soil-rhizosphere-root continuum.

The assembly process of root-associated microbes is influenced by many factors, such as root exudates, the plant immune system, and soil physicochemical properties. Revealing the dynamic ecological processes of plant root-associated microbiome assembly will facilitate the utilization of possible approaches to adjust the microbial community to provide beneficial functions for soil and plant health. These environmentally friendly approaches can be applied to agricultural practices to avoid excessive fertilizer additions (1, 57). Previous studies have indicated that various compartment niches are driven by distinct assembly processes during the development of plants (39). Our results showed that deterministic processes played a more significant role in the assembly of bacterial communities in the rhizosphere and endosphere than in the assembly of fungal communities. Compared with fungi, most bacteria have a narrow tolerance range for growth and are more sensitive to variations in microenvironmental responses (32, 58, 59). The rhizosphere is a carbon-rich compartment niche as a consequence of root exudation or rhizodeposition, providing a more diverse microenvironment for microorganisms (60). There was a significant correlation between bacterial communities and the phosphorus content of the rhizosphere soil (Fig. S5). To meet the requirement for phosphorus, plants secrete low-molecular-weight organic acids in the rhizosphere to increase phosphorus availability, while plants recruit and select for specific microbial communities with phosphorus-solubilizing capacity in the rhizosphere (61). Therefore, it is reasonable that the metabolic activity of roots contributes to the variation in soil physicochemical properties in the microenvironment, which makes the assembly of bacterial communities more susceptible to being driven by deterministic processes. Furthermore, deterministic processes in fungal communities that gradually increased along the soil-rhizosphere-root continuum were observed. Previous studies suggest that in a relatively homogeneous natural tropical forest, fungi are more highly associated with plant phylogeny than bacteria are (62). Simultaneously, specific fungi can form biotrophic relationships with plants (42), particularly with the poorly developed root systems of blueberry, which can form symbiotic associations with specific fungi (56). Therefore, this could be a response to a plant host effect, in which the plants exert a stronger selective effect on the fungal community, contributing to a progressive increase in the influence of deterministic processes from the rhizosphere to the endosphere.

Rich and diverse relationships between bacteria and fungi, such as antagonistic and cooperative relationships, influence the dynamics of microbial communities (63). Our study indicated that higher positive bacterial-fungal interkingdom interactions were observed along the soil-rhizosphere-root continuum. The positive interactions that exist between bacteria and fungi may be due to their mutualistic and commensal ecological interactions (64). In general, recalcitrant organic matter, such as lignin and cellulose, is largely degraded by fungi and releases products like phenolic compounds and water-soluble sugars that are utilized by bacteria (65). Additionally, there are certain bacteria that can facilitate the formation of ectomycorrhizae, colonize the surface of fungal hyphae, and benefit from the exudates of the fungi (66). Several studies have also identified partial bacterial taxa that interact with fungi (64). It has been suggested that the greater numberof positive relationships observed in the rhizosphere of Astragalus mongholicus implied a higher degree of cooperative interactions, which may contribute to plant resistance against abiotic and biotic stresses (61). Host plants and root-associated microbial communities are an evolutionary unit of common interest, and root-associated microbes can regulate host root traits through belowground nutrient acquisition strategies of nutrient scavenging and nutrient mining. Plants reallocate the release of root exudates and the recruitment of beneficial microorganisms, respectively, to ultimately achieve nutrient acquisition and plant growth (67, 68). Previous studies show that the synergistic interaction of arbuscular mycorrhizal fungi and nitrogen-fixing rhizobial bacteria can provide different limiting nutrients for plant growth (69). This indicates that the association between bacterial and fungal communities acts as an essential driver of plant health and that plants have the potential to exploit the positive interactions of bacterial and fungal communities to meet functional requirements. In addition, interactions within and among fungal and bacterial communities are important for sustaining multiple ecosystem functions (70). They are the basic consumer trophic level of the soil food web and are therefore influenced by higher trophic levels of soil biota (8). As major soil protists, the high association of certain cercozoan taxa with bacteria and fungi suggests a hierarchical structure of trophic networks in which predators gain dominance over other predators (71). However, positively correlated cooperative or synthetic relationships between bacteria and fungi are likely to be a shift toward resistant prey organisms in trophic interactions with protists (72, 73).

### Keystone taxa of the blueberry root-associated microbiome and their ecological functions.

The keystone microbial species were positively associated with plant productivity and could be considered potential candidates for manipulating microorganisms to promote plant production and reduce fertilizer inputs (8). Our results showed that there were 19 and 12 keystone taxa belonging to *Proteobacteria* and *Actinobacteria* (Table S8), respectively, that had high relative abundances in each compartment niche of blueberry roots. Previous studies have shown that a positive correlation between the relative abundance of *Proteobacteria* and the rate of nitrogen application was observed during long-term nitrogen fertilization, suggesting that *Proteobacteria* were strongly affected by nitrogen addition (74, 75). The increased abundance of *Proteobacteria* could be attributed to the copiotroph life history strategy, which favors the nutrient-sufficient habitat (74). Thus, the high relative abundance of *Proteobacteria* could promote nitrogen consumption and turnover, which may potentially participate in soil nitrogen cycling and contribute to the nitrogen fixation effect (76). Notably, *Actinobacteria* was the dominant taxon of the bacterial community in the root endosphere. Members of *Actinobacteria* have been noted to play a crucial role in plant disease suppression by producing secondary metabolites like antibiotics and antifungal compounds (77, 78). Therefore, it is possible that bacterial communities were recruited from rhizosphere soils and bulk soils for the functions required by plants (79). On these bases, we speculate that these keystone taxa may play critical ecological roles in promoting soil nutrient cycling and enhancing the abiotic or biotic stress tolerance of host plants.

Furthermore, our results indicated that the class *Sordariomycetes* was an abundant fungal taxon in the bulk soil, rhizosphere, and endosphere but gradually declined along the soil-rhizosphere-root continuum, which is consistent with previous studies showing that members of *Sordariomycetes* were the predominant fungal taxa in the soil (39) and that their abundance was positively related to the nitrogen concentration (80, 81). Additionally, 7 keystone taxa in *Eurotiales* were observed in the bulk soil and rhizosphere (Table S8). It was reported that most species in the order *Eurotiales* in the class *Eurotiomycetes* possess N_2_O-producing activity (82). Moreover, our results indicated that fungal keystone taxa in *Helotiales*, *Chaetothyriales*, and *Sebacinales* were more abundant in the rhizosphere and endosphere (Table S8), where these orders contain the known ERM fungi (25). The genus *Oidiodendron* was predominant in the endosphere and belongs to the ERM fungi, which play a critical role in promoting blueberry root growth and development, particularly in nutrient-deprived habitats (15, 18, 19). Previous studies have shown that ERM fungi have the ability to reciprocally exchange carbon and phosphorus, along with the ability to decompose complex organic compounds to enhance the nutrient acquisition and fitness of plants (83, 84). Thus, keystone fungal species may potentially play an ecological role as decomposers, facilitating rhizosphere turnover of plant-derived carbon and providing additional organic nutrients to plants. Collectively, our results suggest that plants can recruit specific microbes to satisfy the functions that are required by host plants in different compartment niches (79). While the identification of keystone taxa has provided insight into the contributions of microorganisms to improve the productivity and health of host plants, the beneficial functions of microorganisms for plants need to be further explored.

### Conclusions.

This study demonstrates that root compartment niches strongly influence the diversity, community compositions, and co-occurrence network patterns of the blueberry root-associated microbiome. Root compartment niches predominate over host cultivars of blueberry in shaping the root-associated microbiome. Totals of 33 and 20 distinct biomarkers, respectively, were identified in the bacterial and fungal communities of different compartment niches. As host effects increased, deterministic processes gradually increased along the soil-rhizosphere-root continuum for both bacterial and fungal communities. The complexity of bacterial-fungal interkingdom interactions decreased along the soil-rhizosphere-root continuum, while positive interactions gradually dominated in the co-occurrence networks. Functional predictions showed that bacterial communities in the rhizosphere had higher capacities for cellulolysis, and the ecological guilds of fungal communities in the root endosphere, rhizosphere, and bulk soil were ericoid mycorrhizae, soil saprotrophs, and plant pathogens, respectively. These findings provide insight into the variation and assembly processes in different root compartment niches of blueberry, as well as the interkingdom interactions and potentially beneficial functions, which may contribute to the manipulation of microorganisms to promote productivity and enhance plant tolerance to biotic/abiotic stresses.

## MATERIALS AND METHODS

### Study site and sample collection.

The study site was located in the Blueberry Germplasm Resource Station (31°60′N, 119°20′E), Lishui, Nanjing, Jiangsu Province, China. The soil type is a Hydragric Anthrosol, according to the World Reference Base for Soil Resources classification. The sampling area was established in 2013 and covers an area of approximately 30,000 m^2^ with flat topography. Three blueberry cultivars were used, including rabbiteye blueberry, northern highbush blueberry, and southern highbush blueberry. The selected plants were grown with consistent and conventional management practices from the same field. Six sampling sites were selected randomly for each blueberry cultivar, and each sampling site was 15 by 15 m^2^. For representative samples, a multipoint composite sampling method was adopted, with each composite sample used for subsequent analysis representing 12 individual samples. Each rhizosphere soil and root sample was collected randomly at a 10-cm depth with a clean spade from three individual blueberry plants at the same growth stage from four directions in October 2021 (85). Bulk soil samples were collected from sampling sites for each blueberry cultivar in the absence of planting and fertilization. Samples were stored at low temperatures and delivered immediately to the laboratory. For rhizosphere soil samples, soil closely adhered to the roots was collected using a sterilized brush and then sieved through a 2-mm sieve. For root endosphere samples, the roots were rinsed three times using sterile water and then transferred into 50-mL sterilized tubes and sonicated for 3 min at 60 Hz (sonication for 30 s, break for 30 s, 3 cycles) to clear the microbes from the rhizoplane. After sonication, the roots were transferred to 2-mL sterile centrifuge tubes, flash-frozen with liquid nitrogen, and then stored in an ultralow-temperature refrigerator (79). Individual soil samples were kept at −20°C and −80°C for soil physiochemical analysis and microbial community analysis, respectively.

### Illumina sequencing and bioinformatic analysis.

DNA samples were extracted from 0.5-g amounts of bulk soil, rhizosphere soil, and root endosphere samples using FastDNA spin kits (MP Biomedicals, Santa Ana, CA, USA) following the manufacturer’s instructions. The V5-V7 region of the bacterial 16S rRNA gene and the internal transcribed spacer 1 (ITS1) region of the fungal rRNA gene were amplified. The PCR amplification was sequenced on the Illumina MiSeq PE300 platform (Illumina, San Diego, USA). Detailed DNA extraction and amplification methods are described in the supplemental material. The accession number of the raw sequences is PRJNA907196 (NCBI Sequence Read Archive).

The raw sequences were denoised and sorted, the operational taxonomic units (OTUs) clustered using UPARSE (version 7.1) at a similarity of 97%, and chimeric sequences identified and removed using UCHIME. The taxonomy of each sequence was based on our previous methods using the UNITE (version 8.0, http://unite.ut.ee/index.php) and SILVA (version 13.8, http://www.arb-silva.de) databases for fungi and bacteria, respectively (85). In total, 2,162,807 bacterial and 2,597,238 fungal sequence reads were acquired and clustered into 4,470 and 3,856 OTUs, respectively.

### Statistical analysis.

Alpha diversity indices (Chao1 and Shannon) were calculated using Mothur software (version 1.30.2), and beta diversity was assessed by calculating the Bray-Curtis dissimilarity matrix and then ordinated using nonmetric multidimensional scaling analysis (NMDS) by QIIME software (version 1.9.1) (86). Analysis of similarity (ANOSIM) was conducted to determine differences between blueberry compartment niches (87). Two-way PERMANOVA was used to test the relative contributions of various factors to community dissimilarity. Linear discriminant analysis (LDA) effect size (LEfSe) was performed (LDA score of >4.0, *P *< 0.05) in order to identify the biomarkers within the different compartment niches.

The beta nearest-taxon index (βNTI) was calculated by a null model (999 randomizations) to assess the relative importance of deterministic processes (|βNTI| above 2) and stochastic processes (|βNTI| below 2) in driving microbiome assembly (88). These processes were categorized based on the βNTI and Bray-Curtis-based Raup-Crick index (RC_Bray_) values into (i) heterogeneous selection, (ii) homogeneous selection, (iii) dispersal limitation, (iv) homogenizing dispersal, and (v) undominated ecological processes according to (i) βNTI above +2, (ii) βNTI below −2, (iii) |βNTI| below 2 and RC_Bray_ above 0.95, (iv) |βNTI| below 2 and RC_Bray_ below −0.95, and (v) |βNTI| below 2 and |RC_Bray_| below 0.95, respectively (39, 89).

Microbial community co-occurrence networks and interkingdom co-occurrence network analysis were constructed using OTUs with relative abundances of >0.5%. A valid co-occurrence correlation between OTUs was identified as statistically significant at a Spearman’s coefficient of *r *> 0.7 or *r* < −0.7 and (*P* value of <0.01). The *P* values were modified by Benjamini-Hochberg’s false discovery rate method for multiple-testing corrections (90). The co-occurrence network analysis was conducted with iGraph and Hmisc in R (91, 92) and visualized with Gephi software (version 0.9.3). Topological characteristics were applied to characterize the complexity of interactions and network structures of patterns among bacteria and fungi (93). The functional profiles of bacteria and fungi were predicted using FAPROTAX (version 1.1) and FUNGuild (version 1.0), respectively, and analyzed at https://cloud.majorbio.com/.

### Data availability statement.

The data that support the findings of this study are available from the National Center for Biotechnology Information (NCBI) Sequence Read Archive (http://trace.ncbi.nlm.nih.gov/Traces/sra/) under accession number PRJNA907196.
