# Supplementary material for: Root Niches of Blueberry Imprint Increasing Bacterial-Fungal Interkingdom Interactions along the Soil-Rhizosphere-Root Continuum
Source: Microbiol Spectr. 2023 May 24;11(3):e05333-22. doi: 10.1128/spectrum.05333-22 (PMC10269492; doi:10.1128/spectrum.05333-22)
Supplement: Supplemental file 6 — Supplemental material. Download spectrum.05333-22-s0001.docx, DOCX file, 0.07 MB [file spectrum.05333-22-s0001.docx]

**Supplementary** **Information**

**Ecological niches shape the assembly and interaction network of blueberry root-associated microbial communities**

Jilu Che^a^, Yaqiong Wu^b*^, Hao Yang^a^, Shaoyi Wang^a^, Wenlong Wu^b^, Lianfei Lyu^b^, Xiaomin Wang^b^, Weilin Li^a*^

^a^Co-Innovation Center for Sustainable Forestry in Southern China, Nanjing Forestry University, 159 Longpan Road, Nanjing 210037, China, [jlche@njfu.edu.cn](mailto:jlche@njfu.edu.cn), ﻿[yanghao_19940720@163.com](mailto:yanghao_19940720@163.com), [aszx1999@126.com](mailto:aszx1999@126.com), wlli@njfu.edu.cn

^b^Institute of Botany, Jiangsu Province and Chinese Academy of Sciences (Nanjing Botanical Garden Mem. Sun Yat-Sen), Jiangsu Key Laboratory for the Research and Utilization of Plant Resources, Qian Hu Hou Cun No. 1, Nanjing 210014, China, [ya_qiong@126.com](mailto:ya_qiong@126.com), [1964wwl@163.com](mailto:1964wwl@163.com), njbglq@163.com, xmwang525@163.com

***Corresponding authors**

Yaqiong Wu, Weilin Li

E-mails: ya_qiong@126.com, wlli@njfu.edu.cn

**Content**

Soil physiochemical analysis

DNA extraction, Illumina sequencing and ﻿bioinformatic analysis

Processing of sequencing data

**Figures**

Figure S1. The relative abundance of major fungal taxa on orders level present in the rhizosphere of blueberry at different ages.

Figure S2. Linear discriminant analysis effect size (LEfSe) analysis of differentially abundant (LDA threshold score ≥ 4.0) phylum, class, and order of bacteria (A) and fungi (B) of blueberry root-associated microbiomes in different compartment niches.

Figure S3. Co-occurrence network analysis of bacterial and fungal communities in different compartment niches of blueberry.

Figure S4. Heatmaps of the relative abundances of functional groups in each compartment niches of blueberry.

Figure S5. Mantel test analysis between soil physiochemical variables and microbial community composition of blueberry in the bulk soil (A) and rhizosphere (B).

**Tables**

Table S1. The effect of multiple factors on the variation of blueberry root-associated microbial community composition based on two-way PERMANOVA analysis.

Table S2. Relative contribution of ecological processes in microbiome assembly based on the both βNTI and Bray-Curtis-based Raup-Crick Index (RCBray) values.

Table S3. Topological features of co-occurrence network of bacterial-fungal interkingdom interactions in each compartment niche of blueberry.

Table S4. Topological features of co-occurrence network of bacterial communities in each compartment niche of blueberry.

Table S5. ﻿Topological features of co-occurrence network of fungal communities in each compartment niche of blueberry.

Table S6. The relative abundance of multiple correlations between bacterial and fungal taxa in interkingdom networks at different compartment niches of blueberry.

Table S7. The keystone bacterial taxa in interkingdom networks at different compartment niches of blueberry.

Table S8. The keystone fungal taxa in interkingdom networks at different compartment niches of blueberry.

Table S9. Relative abundances of functional groups in each compartment niches of blueberry.

Table S10. Soil parameters of blueberries in the bulk soil and rhizosphere soil.

**Soil physiochemical analysis**

The automatic elemental analyzer (PerkinElmer 2400 Series II, USA) was used for the determination of soil total ﻿nitrogen (TN) and total carbon (TC) content (Tan et al., 2022). Total potassium (TK) and total phosphorus (TP) were digested with HNO_3_- HNO_3_-HF-HClO_4_, and ﻿the available potassium (AK) and available phosphorus (AP) were extracted with HCl-H_2_SO_4_ and ammonium acetate, respectively. TP and AP were determined by an atomic absorption spectrometer (PerkinElmer PinAAcle 900T, USA), and TK and AK were determined by an ultraviolet spectrophotometer, respectively (Jiang et al., 2017). ﻿Soil ammonium-nitrogen (NH_4_^+^-N) and nitrate-nitrogen (NO_3_^−^-N) were measured by ultraviolet spectrophotometer (Shimadzu UVmini-1285, Japan) after extraction with 2 M KCl (Huang et al., 2019). Soil organic matter (SOM) content was measured by the potassium dichromate oxidation method (Yu et al., 2020). Soil pH value was measured by glass electrode in a soil-water solution (w/v) at a fresh soil to water ratio of 1:2.5. Moisture content was measured by oven-drying at 105 ℃ for 48 h.

**DNA extraction, Illumina sequencing and ﻿bioinformatic analysis**

A Nanodrop 2000 UV‒vis spectrophotometer (Thermo Scientific, Wilmington, USA) was used to determine DNA purity and concentration, and DNA quality was checked with 1% agarose gel electrophoresis. The primers 779F (5’-AACMGGATTAGATACCCKG-3’) and 1193R (5’-ACGTCATCCCCACCTTCC-3’), and ITS1F (5’-CTTGGTCATTTAGAGGAAGTAA-3’) and ITS2R (5’-GCTGCGTTCTTCATCGATGC-3’), were carried out for the amplification of the V5-V7 region of bacterial 16S rRNA gene and the ITS1 region of fungal rRNA gene, respectively. The V5-V7 hypervariable regions of the bacterial 16S rRNA gene and ITS1 of the fungal rRNA gene were amplified with primers by thermocycler PCR system (GeneAmp 9700, ABI, USA). The PCR reactions were conducted using the following program: 3 min of denaturation at 95 °C, 27 cycles of 30 s at 95 °C, 30s for annealing at 55 °C, and 45 s for elongation at 72 °C, and a final extension at 72 °C for 10 min. PCR reactions were performed in triplicate 20 μL mixture containing 4 μL of 5 × FastPfu Buffer, 2 μL of 2.5 mM dNTPs, 0.8 μL of each primer (5 μM), 0.4 μL of FastPfu Polymerase and 10 ng of template DNA. The resulted PCR products were extracted from a 2% agarose gel and further purified using the AxyPrep DNA Gel Extraction Kit (Axygen Biosciences, Union City, CA, USA) and quantified using QuantiFluor TM -ST (Promega, USA) according to the manufacturer’s protocol.

**Processing of sequencing data**

Raw fastq files were demultiplexed, quality-filtered by Trimmomatic and merged by FLASH with the following criteria: (i) The reads were truncated at any site receiving an average quality score <20 over a 50 bp sliding window. (ii) Primers were exactly matched allowing 2 nucleotide mismatching, and reads containing ambiguous bases were removed. (iii) Sequences whose overlap longer than 10 bp were merged according to their overlap sequence.

Figure S1. The relative abundance of major fungal taxa on orders level present in the rhizosphere of blueberry at different ages.

Figure S2. Linear discriminant analysis effect size (LEfSe) analysis of differentially abundant (LDA threshold score ≥ 4.0) phylum, class, and order of bacteria (A) and fungi (B) of blueberry root-associated microbiomes in different compartment niches.

Figure S3. Co-occurrence network analysis of bacterial and fungal communities in different compartment niches of blueberry. The networks are colored based on the modularity class of bacterial (A) and fungal (B) communities. Connections indicated significant (p < 0.01) correlations, which were divided into positive (Spearman’s p > 0.7; red) or negative (Spearman’s p < -0.7; green) correlations. The size of each node is proportional to the degree of the OTUs.

Figure S4. Heatmaps of the relative abundances of functional groups in each compartment niches of blueberry. The color from blue to red represents a relative abundance from low to high.

Figure S5. Mantel test analysis between soil physiochemical variables and microbial community composition of blueberry in the bulk soil (A) and rhizosphere (B). The bacterial and fungal community compositions were related to each soil physiochemical parameters by Mantel tests. Edge width corresponds to Mantel’s r statistic for the corresponding distance correlations, and edge color denotes the statistical significance based on 999 permutations. Correlation comparisons of Pearson’s correlation coefficients for soil physiochemical parameters are shown as a color gradient. OM, organic matter; TC, total carbon content; TN, total nitrogen content; TP, total phosphorus content; TK, total potassium content; NH_4_^+^-N, nitrate nitrogen; NO_3_^−^-N, ammonium nitrogen; AP, available P content; AK, available K content.

Table S1 The effect of multiple factors on the variation of blueberry root-associated microbial community composition based on two-way PERMANOVA analysis.

|  | Variables | *F* | *R^2^* | *p* |
| --- | --- | --- | --- | --- |
| Bacterial community | Compartment niches | 25.245 | 0.564 | 0.001 |
|  | Host cultivars | 3.754 | 0.161 | 0.001 |
| Fungal  community | Compartment niches | 9.974 | 0.339 | 0.001 |
|  | Host cultivars | 4.340 | 0.182 | 0.001 |

Table S2 Relative contribution of ecological processes in microbiome assembly based on the both βNTI and Bray-Curtis-based Raup-Crick Index (RCBray) values.

|  | Bacteria | | | Fungi | | |
| --- | --- | --- | --- | --- | --- | --- |
|  | Bulk soil | Rhizosphere soil | Root endosphere | Bulk soil | Rhizosphere soil | Root endosphere |
| determinism | 0% | 66% | 60% | 7% | 12% | 24% |
| stochasticity | 100% | 34% | 40% | 93% | 88% | 76% |
| homogeneous selectipn: βNTI < - 2 | 0% | 66% | 55% | 7% | 4% | 24% |
| heterogeneous selection: βNTI > + 2 | 0% | 0% | 5% | 0% | 8% | 0% |
| dispersal limitation: \|βNTI\|< 2 and RCBray > 0.95 | 0% | 0% | 0% | 0% | 0% | 0% |
| homogenizing dispersal: \|βNTI\|< 2 and RCBray < – 0.95 | 7% | 10% | 11% | 7% | 12% | 11% |
| undominated: \|βNTI\|< 2 and \|RCBray\|< 0.95 | 93% | 24% | 29% | 87% | 75% | 65% |

Note: The βNTI values were calculated using null model, and |βNTI| ≥ 2 and |βNTI| < 2 represent dominant determinism and stochasticity in driving microbiome assembly, respectively. The five ecological processes including heterogeneous selection (βNTI > + 2), homogeneous selection (βNTI < − 2), dispersal limitation (|βNTI| < 2 and RCBray > 0.95), homogenizing dispersal (|βNTI| < 2 and RCBray < – 0.95), and undominated (|βNTI| < 2 and |RCBray| < 0.95).

Table S3 ﻿Topological features of co-occurrence network of bacterial-fungal interkingdom interactions in each compartment niche of blueberry.

| Network metrics | Bulk soil | Rhizosphere soil | | | Root endosphere | | |
| --- | --- | --- | --- | --- | --- | --- | --- |
|  |  | RB | NB | SB | RB | NB | SB |
| Number of total nodes | 841 | 467 | 477 | 496 | 153 | 115 | 170 |
| Number of bacteria nodes | 521 | 325 | 314 | 316 | 114 | 89 | 129 |
| Number of fungi nodes | 320 | 142 | 163 | 180 | 39 | 26 | 41 |
| Number of total edges | 2702 | 908 | 1100 | 794 | 139 | 90 | 151 |
| Number of total bacteria-bacteria correlations (TBB) | 1013 | 384 | 454 | 323 | 79 | 63 | 92 |
| Number of bacteria-bacteria positive correlations (PBB) | 639 | 201 | 259 | 183 | 60 | 35 | 55 |
| Number of bacteria-bacteria negative correlations (NBB) | 374 | 183 | 195 | 140 | 19 | 28 | 37 |
| Number of total fungi-fungi correlations (TFF) | 1035 | 108 | 144 | 157 | 10 | 8 | 27 |
| Number of fungi-fungi positive correlations (PFF) | 994 | 77 | 112 | 124 | 0 | 6 | 0 |
| Number of fungi-fungi negative correlations (NFF) | 41 | 31 | 32 | 33 | 10 | 2 | 27 |
| Number of total bacteria-fungi correlations (TBF) | 654 | 416 | 502 | 314 | 50 | 19 | 32 |
| Number of bacteria-fungi positive correlations (PBF) | 384 | 240 | 307 | 164 | 37 | 12 | 20 |
| Number of bacteria-fungi negative correlations (NBF) | 270 | 176 | 195 | 150 | 13 | 7 | 12 |
| Average degree (*avgK*) | 6.426 | 3.889 | 4.612 | 3.202 | 1.817 | 1.565 | 1.776 |
| Graph Density | 0.008 | 0.008 | 0.01 | 0.006 | 0.012 | 0.014 | 0.011 |
| Modularity (M) | 0.959 | 0.957 | 0.97 | 0.978 | 0.969 | 0.956 | 0.958 |

Note: The RB, NB, and SB represent rabbiteye blueberry, northern highbush blueberry, and southern highbush blueberry, respectively.

Table S4 ﻿Topological features of co-occurrence network of bacterial communities in each compartment niche of blueberry.

| Network metrics | Bulk soil | Rhizosphere soil | | | Root endosphere | | |
| --- | --- | --- | --- | --- | --- | --- | --- |
|  |  | RB | NB | SB | RB | NB | SB |
| Number of nodes | 486 | 298 | 284 | 284 | 101 | 75 | 115 |
| Number of edges | 1013 | 389 | 454 | 325 | 79 | 63 | 92 |
| Number of positive correlations | 558 | 224 | 254 | 170 | 63 | 33 | 49 |
| Number of negative correlations | 455 | 165 | 200 | 155 | 16 | 30 | 43 |
| Average degree (*avgK*) | 4.169 | 2.611 | 3.197 | 2.289 | 1.564 | 1.68 | 1.6 |
| Graph Density | 0.009 | 0.009 | 0.011 | 0.008 | 0.016 | 0.023 | 0.014 |
| Modularity (M) | 0.966 | 0.961 | 0.964 | 0.966 | 0.962 | 0.928 | 0.954 |

Note: The RB, NB, and SB represent rabbiteye blueberry, northern highbush blueberry, and southern highbush blueberry, respectively.

Table S5 ﻿Topological features of co-occurrence network of fungal communities in each compartment niche of blueberry.

| Network metrics | Bulk soil | Rhizosphere soil | | | Root endosphere | | |
| --- | --- | --- | --- | --- | --- | --- | --- |
|  |  | RB | NB | SB | RB | NB | SB |
| Number of nodes | 259 | 101 | 126 | 139 | 20 | 13 | 27 |
| Number of edges | 1031 | 108 | 144 | 157 | 10 | 8 | 27 |
| Number of positive correlations | 1008 | 77 | 112 | 124 | 10 | 6 | 27 |
| Number of negative correlations | 23 | 31 | 32 | 33 | 0 | 2 | 0 |
| Average degree (*avgK*) | 7.961 | 2.139 | 2.286 | 2.259 | 1 | 1.231 | 2 |
| Graph Density | 0.031 | 0.021 | 0.018 | 0.016 | 0.053 | 0.103 | 0.077 |
| Modularity (M) | 0.829 | 0.916 | 0.955 | 0.945 | 0.9 | 0.781 | 0.667 |

Note: The RB, NB, and SB represent rabbiteye blueberry, northern highbush blueberry, and southern highbush blueberry, respectively.

Table S6 The relative abundance of multiple correlations between bacterial and fungal taxa in interkingdom networks at different compartment niches of blueberry.

| Network metrics | Bulk soil | Rhizosphere soil | Root endosphere |
| --- | --- | --- | --- |
| Percentage of bacteria nodes | 62.00% | 66.40% | 75.90% |
| Percentage of fungi nodes | 38.00% | 34.60% | 24.10% |
| Average degree (avgK) of bacteria | 5.15 | 3.73 | 1.71 |
| Average degree (avgK) of fungi | 8.5 | 4.25 | 3.75 |
| Percentage of total bacteria-bacteria correlations | 37.50% | 41.40% | 62.60% |
| Percentage of total fungi-fungi correlations | 38.30% | 14.90% | 11.30% |
| Percentage of total bacteria-fungi correlations | 24.20% | 43.70% | 26.10% |
| Percentage of bacteria-fungi positive correlations | 58.72% | 57.71% | 68.32% |
| Percentage of bacteria-fungi negative correlations | 41.28% | 42.29% | 31.68% |
| Percentage of total positive correlations | 74.60% | 59.30% | 59.40% |
| Percentage of total negative correlations | 25.40% | 40.70% | 40.60% |

Table S7 The keystone bacterial taxa in interkingdom networks at different compartment niches of blueberry.

|  | name | phylum | class | order | family | genus |
| --- | --- | --- | --- | --- | --- | --- |
| Bulk soil | OTU4148 | Myxococcota | Polyangia | Polyangiales | Sandaracinaceae | unclassified |
|  | OTU738 | Proteobacteria | Alphaproteobacteria | Rhizobiales | Rhizobiaceae | unclassified |
| Rhizosphere soil | OTU2208 | Acidobacteriota | Acidobacteriae | Acidobacteriales | Acidobacteriaceae_Subgroup_1 | Acidipila |
|  | OTU3398 | Acidobacteriota | Acidobacteriae | Acidobacteriales | unclassified | unclassified |
|  | OTU2501 | Acidobacteriota | Acidobacteriae | Solibacterales | Solibacteraceae | Candidatus_Solibacter |
|  | OTU3408 | Acidobacteriota | Acidobacteriae | Acidobacteriales | unclassified | unclassified |
|  | OTU3472 | Acidobacteriota | Acidobacteriae | Acidobacteriales | unclassified | unclassified |
|  | OTU3571 | Acidobacteriota | Acidobacteriae | Acidobacteriales | unclassified | unclassified |
|  | OTU3393 | Actinobacteriota | Actinobacteria | Frankiales | Acidothermaceae | Acidothermus |
|  | OTU2347 | Actinobacteriota | Actinobacteria | Frankiales | Acidothermaceae | Acidothermus |
|  | OTU3287 | Actinobacteriota | Actinobacteria | Frankiales | Frankiaceae | Jatrophihabitans |
|  | OTU4679 | Actinobacteriota | Actinobacteria | Pseudonocardiales | Pseudonocardiaceae | unclassified |
|  | OTU2135 | Actinobacteriota | Actinobacteria | Catenulisporales | Actinospicaceae | Actinospica |
|  | OTU1536 | Actinobacteriota | Actinobacteria | Catenulisporales | Actinospicaceae | Actinospica |
|  | OTU3221 | Actinobacteriota | Actinobacteria | Micrococcales | Microbacteriaceae | Humibacter |
|  | OTU3572 | Actinobacteriota | Actinobacteria | Frankiales | Geodermatophilaceae | Geodermatophilus |
|  | OTU2234 | Chloroflexi | Ktedonobacteria | Ktedonobacterales | Ktedonobacteraceae | G12-WMSP1 |
|  | OTU3697 | Chloroflexi | Ktedonobacteria | Ktedonobacterales | JG30-KF-AS9 | unclassified |
|  | OTU3529 | Chloroflexi | Ktedonobacteria | Ktedonobacterales | Ktedonobacteraceae | FCPS473 |
|  | OTU3217 | Chloroflexi | Ktedonobacteria | Ktedonobacterales | Ktedonobacteraceae | unclassified |
|  | OTU3543 | Chloroflexi | Ktedonobacteria | Ktedonobacterales | JG30-KF-AS9 | norank_JG30-KF-AS9 |
|  | OTU2875 | Gemmatimonadota | Gemmatimonadetes | Gemmatimonadales | Gemmatimonadaceae | unclassified |
|  | OTU2489 | Proteobacteria | Gammaproteobacteria | Burkholderiales | Burkholderiaceae | Ralstonia |
|  | OTU4024 | Proteobacteria | Gammaproteobacteria | Burkholderiales | Comamonadaceae | Rhizobacter |
|  | OTU2277 | Proteobacteria | Alphaproteobacteria | Caulobacterales | Caulobacteraceae | Phenylobacterium |
|  | OTU2140 | Proteobacteria | Gammaproteobacteria | Gammaproteobacteria | unclassified | Acidibacter |
|  | OTU3876 | Proteobacteria | Alphaproteobacteria | Micropepsales | Micropepsaceae | unclassified |
|  | OTU3886 | Proteobacteria | Alphaproteobacteria | Micropepsales | Micropepsaceae | unclassified |
|  | OTU1790 | Proteobacteria | Alphaproteobacteria | Acetobacterales | Acetobacteraceae | unclassified |
|  | OTU2329 | Proteobacteria | Alphaproteobacteria | Elsterales | unclassified | unclassified |
|  | OTU848 | Proteobacteria | Alphaproteobacteria | Rhizobiales | Xanthobacteraceae | unclassified |
|  | OTU3951 | Proteobacteria | Alphaproteobacteria | Acetobacterales | Acetobacteraceae | unclassified |
|  | OTU2081 | Proteobacteria | Alphaproteobacteria | Acetobacterales | Acetobacteraceae | unclassified |
|  | OTU2242 | Proteobacteria | Alphaproteobacteria | Rhizobiales | Xanthobacteraceae | unclassified |
| Endosphere | OTU3746 | Acidobacteriota | Acidobacteriae | Subgroup_2 | unclassified | unclassified |
|  | OTU2187 | Acidobacteriota | Acidobacteriae | Bryobacterales | Bryobacteraceae | Bryobacter |
|  | OTU4643 | Acidobacteriota | Acidobacteriae | Acidobacteriales | Acidobacteriaceae_Subgroup_1 | unclassified |
|  | OTU2171 | Acidobacteriota | Acidobacteriae | Acidobacteriales | Acidobacteriaceae_Subgroup_1 | Occallatibacter |
|  | OTU2181 | Actinobacteriota | Actinobacteria | Catenulisporales | Actinospicaceae | Actinospica |
|  | OTU1536 | Actinobacteriota | Actinobacteria | Catenulisporales | Actinospicaceae | Actinospica |
|  | OTU2134 | Actinobacteriota | Actinobacteria | Micromonosporales | Micromonosporaceae | unclassified |
|  | OTU1765 | Actinobacteriota | Actinobacteria | Bifidobacteriales | Bifidobacteriaceae | Bifidobacterium |
|  | OTU1805 | Bacteroidota | Bacteroidia | Bacteroidales | Rikenellaceae | Alistipes |
|  | OTU3497 | Chloroflexi | JG30-KF-CM66 | unclassified | unclassified | unclassified |
|  | OTU1676 | Firmicutes | Clostridia | Clostridiales | Clostridiaceae | unclassified |
|  | OTU1950 | Firmicutes | Clostridia | Oscillospirales | Ruminococcaceae | Faecalibacterium |
|  | OTU2685 | Proteobacteria | Alphaproteobacteria | Rhizobiales | Xanthobacteraceae | Bradyrhizobium |
|  | OTU2446 | Proteobacteria | Alphaproteobacteria | Rhizobiales | Xanthobacteraceae | unclassified |
|  | OTU2204 | Proteobacteria | Alphaproteobacteria | Acetobacterales | Acetobacteraceae | unclassified |
|  | OTU2935 | Proteobacteria | Gammaproteobacteria | Xanthomonadales | Rhodanobacteraceae | Chujaibacter |
|  | OTU1520 | Proteobacteria | Alphaproteobacteria | Caulobacterales | Caulobacteraceae | Asticcacaulis |
|  | OTU2348 | Proteobacteria | Alphaproteobacteria | unclassified | unclassified | unclassified |

Table S8 The keystone fungal taxa in interkingdom networks at different compartment niches of blueberry.

|  | name | phylum | class | order | family | genus |
| --- | --- | --- | --- | --- | --- | --- |
| Bulk soil | ASV951 | Ascomycota | Dothideomycetes | Pleosporales | Sporormiaceae | Westerdykella |
|  | ASV649 | Ascomycota | Dothideomycetes | Pleosporales | Lentitheciaceae | Poaceascoma |
|  | ASV930 | Ascomycota | Eurotiomycetes | unclassified Eurotiomycetes | unclassified | unclassified |
|  | ASV1323 | Ascomycota | Eurotiomycetes | Eurotiales | Trichocomaceae | Talaromyces |
|  | ASV630 | Ascomycota | Eurotiomycetes | Eurotiales | Aspergillaceae | Aspergillus |
|  | ASV4027 | Ascomycota | Eurotiomycetes | Eurotiales | Aspergillaceae | Aspergillus |
|  | ASV2753 | Basidiomycota | Geminibasidiomycetes | Geminibasidiales | Geminibasidiaceae | Geminibasidium |
|  | ASV4228 | Basidiomycota | Geminibasidiomycetes | Geminibasidiales | Geminibasidiaceae | Geminibasidium |
|  | ASV1294 | Glomeromycota | Glomeromycetes | Glomerales | Glomeraceae | unclassified |
|  | ASV469 | Glomeromycota | Glomeromycetes | Glomerales | Glomeraceae | unclassified |
|  | ASV943 | Kickxellomycota | Kickxellomycetes | Kickxellales | Kickxellaceae | Ramicandelaber |
|  | ASV856 | Kickxellomycota | Kickxellomycetes | Kickxellales | Kickxellaceae | Ramicandelaber |
|  | ASV1252 | Kickxellomycota | Kickxellomycetes | Kickxellales | Kickxellaceae | Ramicandelaber |
|  | ASV1029 | Ascomycota | Leotiomycetes | Helotiales | Helotiaceae | Tetracladium |
|  | ASV572 | Olpidiomycota | Olpidiomycetes | Olpidiales | Olpidiaceae | Olpidium |
|  | ASV599 | Glomeromycota | Paraglomeromycetes | GS24 | unclassified_GS24 | unclassified_GS24 |
|  | ASV1243 | Ascomycota | Pezizomycetes | Pezizales | unclassified | unclassified |
|  | ASV836 | Ascomycota | Sordariomycetes | Sordariales | Lasiosphaeriaceae | Schizothecium |
|  | ASV910 | Ascomycota | Sordariomycetes | Xylariales | Xylariales_fam_Incertae_sedis | Neoidriella |
| Rhizosphere soil | ASV4440 | Basidiomycota | Agaricomycetes | Auriculariales | unclassified | unclassified |
|  | ASV3792 | Calcarisporiellomycota | Calcarisporiellomycetes | Calcarisporiellales | Calcarisporiellaceae | Calcarisporiella |
|  | ASV2308 | Ascomycota | Eurotiomycetes | Eurotiales | Aspergillaceae | Aspergillus |
|  | ASV2985 | Ascomycota | Eurotiomycetes | Eurotiales | Aspergillaceae | Penicillium |
|  | ASV2363 | Ascomycota | Eurotiomycetes | Eurotiales | Aspergillaceae | Aspergillus |
|  | ASV2440 | Ascomycota | Eurotiomycetes | Eurotiales | Aspergillaceae | Aspergillus |
|  | ASV1693 | Ascomycota | Eurotiomycetes | Chaetothyriales | unclassified | unclassified |
|  | ASV1693 | Ascomycota | Eurotiomycetes | Chaetothyriales | unclassified | unclassified |
|  | ASV3466 | Ascomycota | Eurotiomycetes | Chaetothyriales | unclassified | unclassified |
|  | ASV3828 | Ascomycota | Leotiomycetes | Helotiales | Helotiaceae | Collophora |
|  | ASV1878 | Ascomycota | Sordariomycetes | unclassified | unclassified | unclassified |
|  | ASV1941 | Ascomycota | Sordariomycetes | Sordariales | Chaetomiaceae | Trichocladium |
|  | ASV1446 | Ascomycota | Sordariomycetes | Hypocreales | Cordycipitaceae | Simplicillium |
|  | ASV2286 | Ascomycota | Sordariomycetes | Hypocreales | Hypocreaceae | Trichoderma |
|  | ASV3957 | Basidiomycota | Tremellomycetes | Tremellales | Trimorphomycetaceae | Saitozyma |
|  | ASV3957 | Basidiomycota | Tremellomycetes | Tremellales | Trimorphomycetaceae | Saitozyma |
| Endosphere | ASV2923 | Basidiomycota | Agaricomycetes | Sebacinales | Serendipitaceae | Serendipita |
|  | ASV2818 | Basidiomycota | Agaricomycetes | Agaricales | Clavariaceae | Clavaria |
|  | ASV134 | Basidiomycota | Agaricomycetes | Agaricales | Agaricaceae | Chlorophyllum |
|  | ASV1694 | Ascomycota | Archaeorhizomycetes | Archaeorhizomycetales | Archaeorhizomycetaceae | Archaeorhizomyces |
|  | ASV3037 | Ascomycota | Eurotiomycetes | Chaetothyriales | Herpotrichiellaceae | unclassified |
|  | ASV1684 | Ascomycota | Leotiomycetes | Helotiales | Helotiaceae | Meliniomyces |
|  | ASV4472 | Mortierellomycota | Mortierellomycetes | Mortierellales | Mortierellaceae | Mortierella |
|  | ASV2098 | Ascomycota | Sordariomycetes | unclassified | unclassified | unclassified |
|  | ASV906 | Ascomycota | Sordariomycetes | Hypocreales | Nectriaceae | Gibberella |

Table S9 Relative abundances of functional groups in each compartment niches of blueberry.

| Functional groups | Bulk soil (%) | Rhizosphere (%) | Endosphere (%) | *p*-value |
| --- | --- | --- | --- | --- |
| Chemoheterotrophy | 24.26 | 33.09 | 38.35 | <0.001 |
| Aerobic chemoheterotrophy | 22.24 | 30.46 | 36.60 | <0.001 |
| Cellulolysis | 0.72 | 14.02 | 8.31 | <0.001 |
| Animal parasites or symbionts | 6.03 | 2.70 | 2.43 | <0.001 |
| Human pathogens all | 5.58 | 2.18 | 2.34 | 0.001 |
| Nitrogen fixation | 0.81 | 3.30 | 3.24 | <0.001 |
| Human pathogens pneumonia | 5.45 | 1.33 | 0.31 | <0.001 |
| Nitrate reduction | 3.13 | 1.29 | 1.00 | 0.001 |
| Fermentation | 1.40 | 2.10 | 1.73 | 0.114 |
| Aromatic compound degradation | 4.00 | 0.53 | 0.28 | <0.001 |
| Nitrogen respiration | 2.05 | 0.36 | 0.95 | 0.001 |
| Nitrate respiration | 2.05 | 0.33 | 0.84 | 0.002 |
| Manganese oxidation | 0.88 | 2.24 | 0.06 | <0.001 |
| Nitrite respiration | 1.79 | 0.34 | 0.94 | 0.001 |
| Ureolysis | 1.09 | 0.63 | 0.24 | 0.001 |
| Predatory or exoparasitic | 1.27 | 0.55 | 0.11 | <0.001 |
| Phototrophy | 1.33 | 0.20 | 0.02 | <0.001 |
| Plastic degradation | 0.92 | 0.57 | 0.00 | <0.001 |
| Nitrite ammonification | 0.50 | 0.06 | 0.92 | <0.001 |
| Photoautotrophy | 1.29 | 0.17 | 0.01 | <0.001 |
| Photoheterotrophy | 1.26 | 0.16 | 0.01 | <0.001 |
| Nitrite denitrification | 1.28 | 0.13 | 0.01 | <0.001 |
| Nitrous oxide denitrification | 1.28 | 0.13 | 0.01 | <0.001 |
| Nitrate denitrification | 1.28 | 0.13 | 0.01 | <0.001 |
| Denitrification | 1.28 | 0.13 | 0.01 | <0.001 |
| Iron respiration | 0.16 | 1.09 | 0.17 | <0.001 |
| Anoxygenic photoautotrophy S oxidizing | 1.22 | 0.13 | 0.01 | <0.001 |
| Anoxygenic photoautotrophy | 1.22 | 0.13 | 0.01 | <0.001 |
| Nitrate ammonification | 0.50 | 0.03 | 0.81 | <0.001 |
| Invertebrate parasites | 0.45 | 0.52 | 0.02 | <0.001 |
| Xylanolysis | 0.20 | 0.49 | 0.02 | <0.001 |
| Ligninolysis | 0.59 | 0.02 | 0.03 | <0.001 |
| Methylotrophy | 0.53 | 0.03 | 0.02 | <0.001 |
| Methanol oxidation | 0.51 | 0.02 | 0.01 | <0.001 |
| Intracellular parasites | 0.10 | 0.24 | 0.08 | 0.005 |
| Chitinolysis | 0.35 | 0.01 | 0.00 | <0.001 |
| Plant pathogen | 0.20 | 0.02 | 0.01 | <0.001 |
| Dark oxidation of sulfur compounds | 0.15 | 0.02 | 0.01 | <0.001 |
| Hydrocarbon degradation | 0.09 | 0.03 | 0.02 | 0.006 |
| Cyanobacteria | 0.07 | 0.04 | 0.00 | <0.001 |
| Oxygenic photoautotrophy | 0.07 | 0.04 | 0.00 | <0.001 |
| Aromatic hydrocarbon degradation | 0.08 | 0.02 | 0.02 | 0.004 |
| Respiration of sulfur compounds | 0.10 | 0.00 | 0.00 | <0.001 |
| Sulfate respiration | 0.09 | 0.00 | 0.00 | <0.001 |
| Dark thiosulfate oxidation | 0.06 | 0.01 | 0.00 | <0.001 |
| Human pathogens septicemia | 0.06 | 0.00 | 0.00 | <0.001 |
| Methanotrophy | 0.01 | 0.02 | 0.00 | 0.004 |
| Chlorate reducers | 0.02 | 0.00 | 0.00 | 0.008 |

Table S10 Soil parameters of ﻿blueberries in the bulk soil and rhizosphere soil.

| Soil parameters | Bulk soil | Rabbiteye blueberry | Northern highbush blueberry | Southern highbush blueberry |
| --- | --- | --- | --- | --- |
| pH | 7.70±0.07a | 4.34±0.01c | 4.41±0.02c | 4.88±0.02b |
| Moisture (%) | 19.17±0.16b | 18.91±0.14bc | 18.76±0.11c | 19.67±0.16a |
| SOM (g/kg) | 14.58±4.48c | 28.24±1.15b | 32.20±1.49b | 40.21±1.50a |
| TC (g/kg) | 7.67±0.15d | 17.47±0.15c | 18.23±0.25b | 22.43±0.21a |
| TN (g/kg) | 1.33±0.15c | 2.2±0.1b | 2.03±0.06b | 2.37±0.06a |
| TP (g/kg) | 0.14±0.01b | 0.37±0.01a | 0.14±0.01b | 0.15±0.01b |
| TK (g/kg) | 13.84±0.07a | 13.34±0.32b | 12.74±0.15c | 12.9±0.26c |
| AP (mg/kg) | 3.77±0.13c | 51.64±2.06a | 8.00±0.08b | 7.29±0.04b |
| AK (mg/kg) | 177.34±0.39d | 551.41±0.98a | 279.14±2.03c | 322.48±13.07b |
| NO_3_^-^-N (mg/kg) | 1.68±0.13c | 28.46±0.51a | 13.53±1.03b | 13.68±0.39b |
| NH_4_^+^-N(mg/kg) | 5.47±0.31d | 6.81±0.61c | 17.78±0.06a | 8.5±0.06b |
| EC (μs/cm) | 116.13±0.91c | 181.27±0.06a | 132.6±1.66b | 104.5±0.30d |

Note: Values for individual groups are the means of the three replicate soil cores (mean ± standard error). Moisture, soil moisture content; SOM, soil organic matter; TN, total nitrogen content; TP, total phosphorus content; NH_4_^+^-N, Nitrate nitrogen; NO_3_ ^−^-N, Ammonium nitrogen; AP, available P content; AK, available K content; Lowercase letters indicate that means of soil property are significantly different (P < 0.05, ANOVA) among groups.
