# Supplementary figures and images for: Root Niches of Blueberry Imprint Increasing Bacterial-Fungal Interkingdom Interactions along the Soil-Rhizosphere-Root Continuum
Source: Microbiol Spectr. 2023 May 24;11(3):e05333-22. doi: 10.1128/spectrum.05333-22 (PMC10269492; doi:10.1128/spectrum.05333-22)

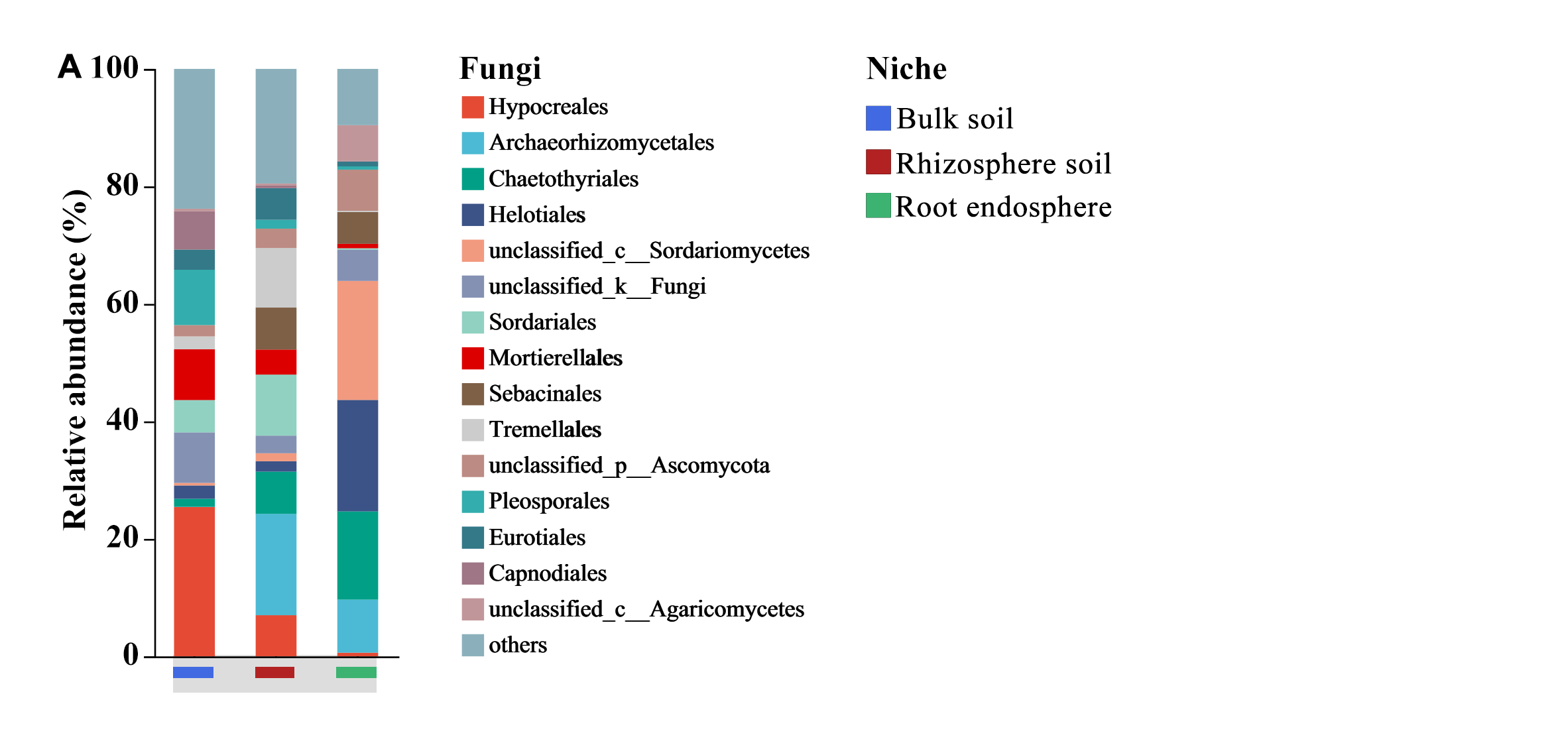

Supplement: Supplemental file 1 — Figure S1. Download spectrum.05333-22-s0002.tif, TIF file, 0.7 MB [file spectrum.05333-22-s0002.tif]

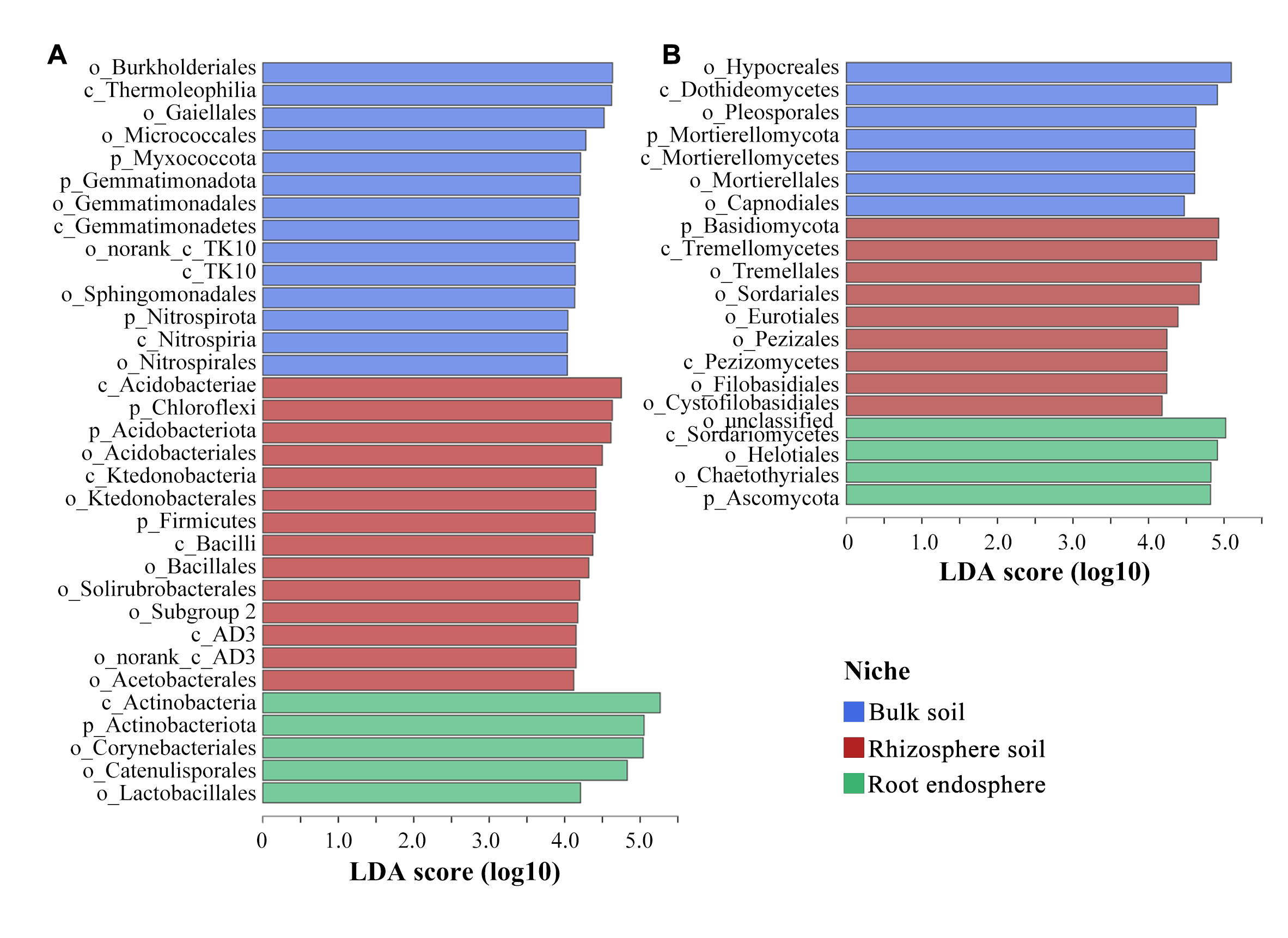

Supplement: Supplemental file 2 — Figure S2. Download spectrum.05333-22-s0003.tif, TIF file, 1.4 MB [file spectrum.05333-22-s0003.tif]

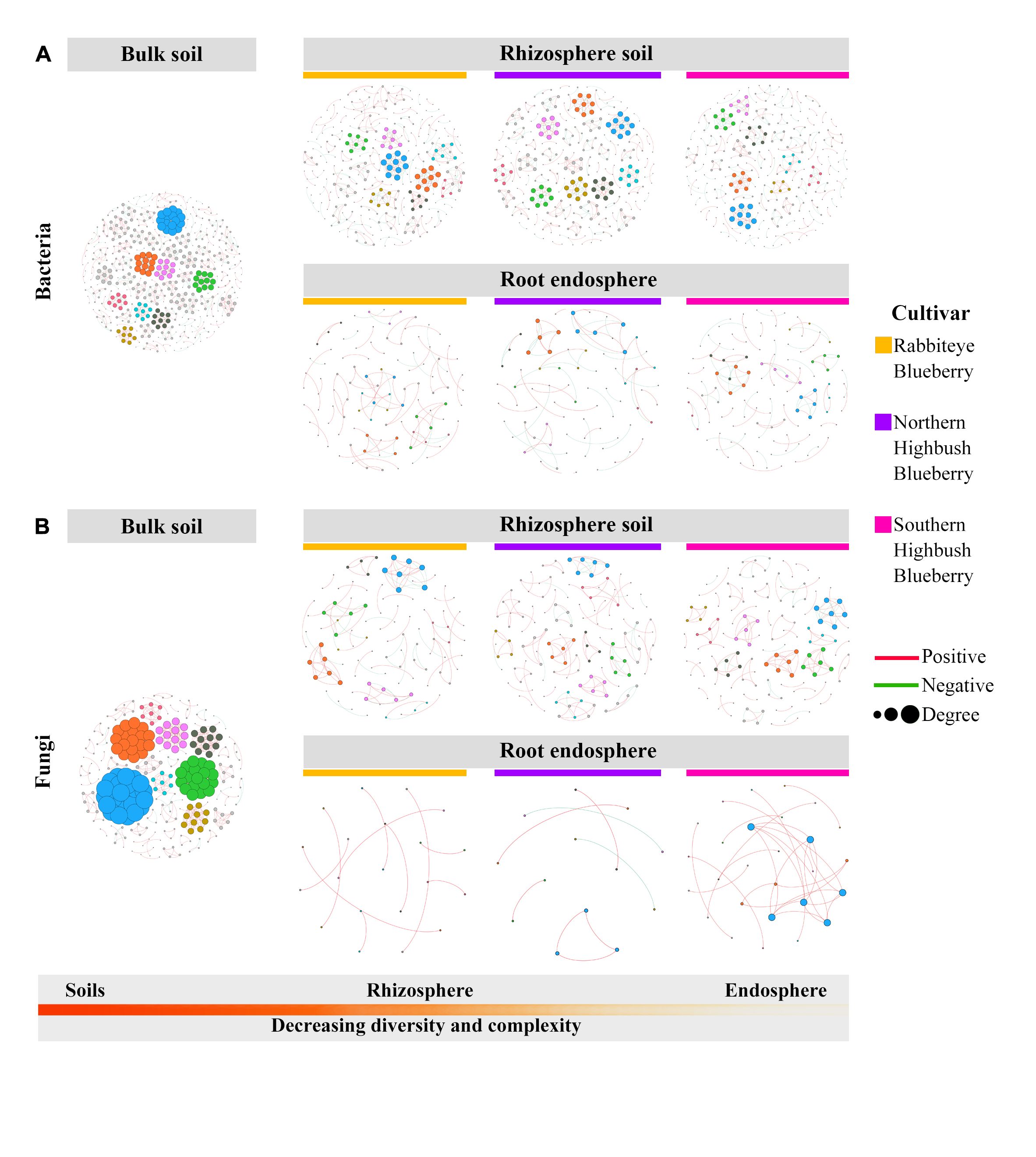

Supplement: Supplemental file 3 — Figure S3. Download spectrum.05333-22-s0004.tif, TIF file, 3.3 MB [file spectrum.05333-22-s0004.tif]

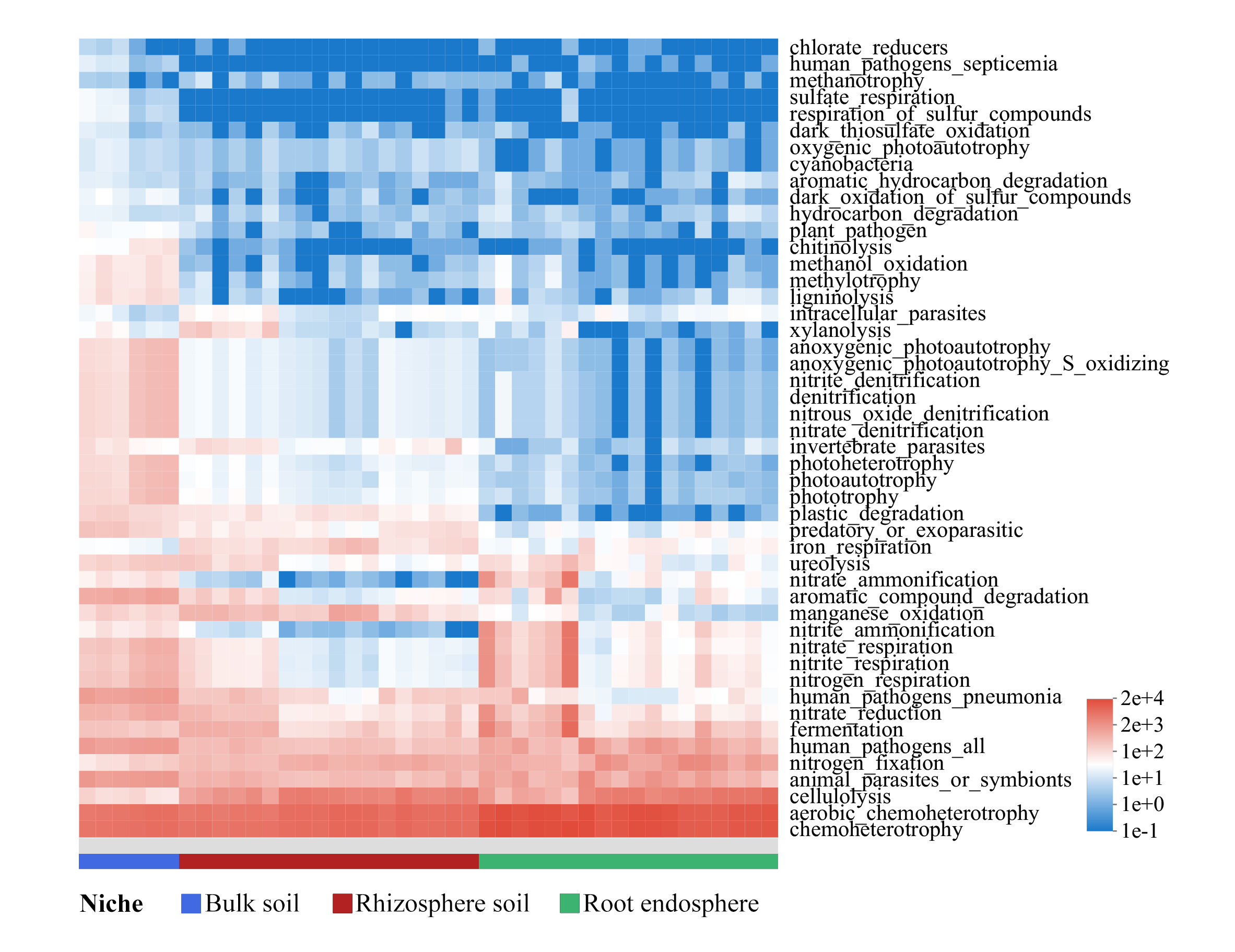

Supplement: Supplemental file 4 — Figure S4. Download spectrum.05333-22-s0005.tif, TIF file, 2.1 MB [file spectrum.05333-22-s0005.tif]

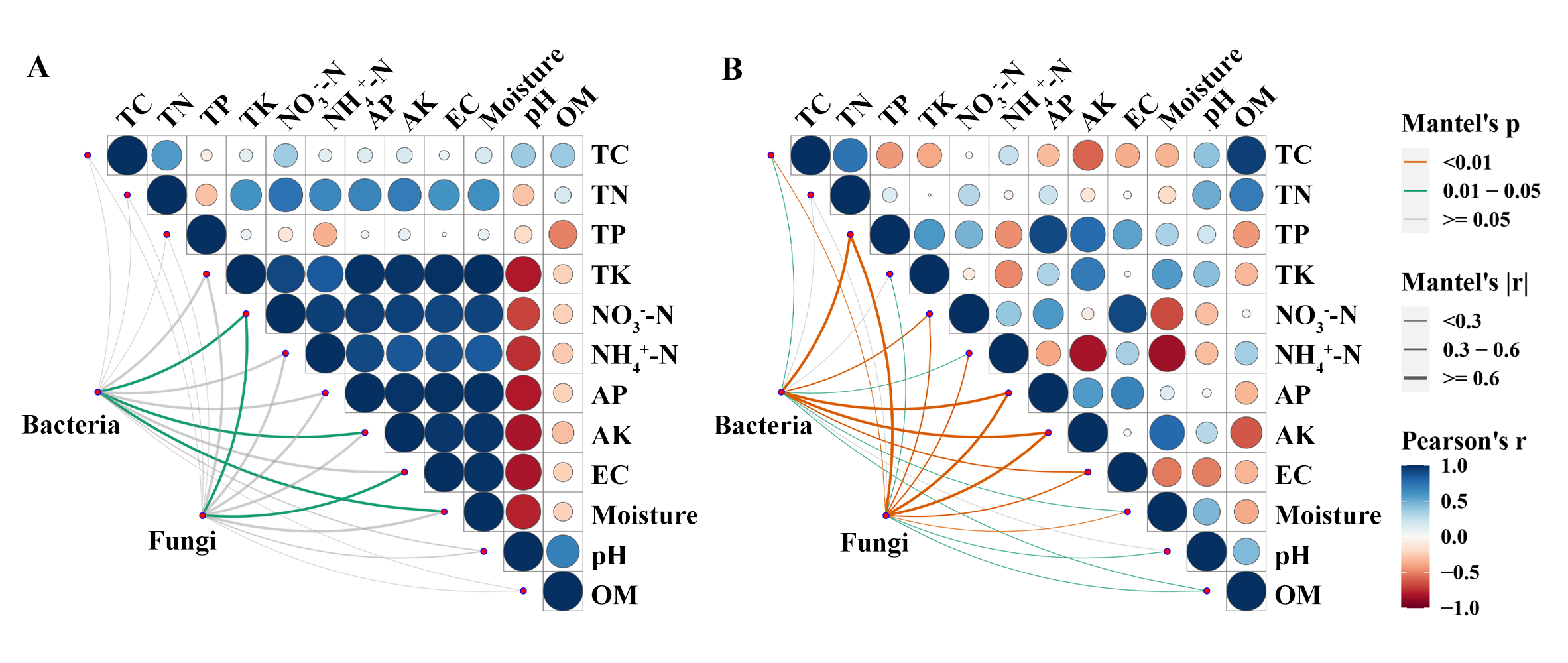

Supplement: Supplemental file 5 — Figure S5. Download spectrum.05333-22-s0006.tif, TIF file, 2.3 MB [file spectrum.05333-22-s0006.tif]
